# Repression of branched-chain amino acid synthesis in *Staphylococcus aureus* is mediated by isoleucine via CodY, and by a leucine-rich attenuator peptide

**DOI:** 10.1371/journal.pgen.1007159

**Published:** 2018-01-22

**Authors:** Julienne C. Kaiser, Alyssa N. King, Jason C. Grigg, Jessica R. Sheldon, David R. Edgell, Michael E. P. Murphy, Shaun R. Brinsmade, David E. Heinrichs

**Affiliations:** 1 Department of Microbiology and Immunology, University of Western Ontario, London, Ontario, Canada; 2 Department of Biology, Georgetown University, Washington, DC, United States of America; 3 Department of Microbiology and Immunology, University of British Columbia, Vancouver, British Columbia, Canada; 4 Department of Biochemistry, University of Western Ontario, London, Ontario, Canada; 5 Department of Microbiology and Immunology, Georgetown University, Washington, DC, United States of America; The University of Texas Health Science Center at Houston, UNITED STATES

## Abstract

*Staphylococcus aureus* requires branched-chain amino acids (BCAAs; isoleucine, leucine, valine) for protein synthesis, branched-chain fatty acid synthesis, and environmental adaptation by responding to their availability via the global transcriptional regulator CodY. The importance of BCAAs for *S*. *aureus* physiology necessitates that it either synthesize them or scavenge them from the environment. Indeed *S*. *aureus* uses specialized transporters to scavenge BCAAs, however, its ability to synthesize them has remained conflicted by reports that it is auxotrophic for leucine and valine despite carrying an intact BCAA biosynthetic operon. In revisiting these findings, we have observed that *S*. *aureus* can engage in leucine and valine synthesis, but the level of BCAA synthesis is dependent on the BCAA it is deprived of, leading us to hypothesize that each BCAA differentially regulates the biosynthetic operon. Here we show that two mechanisms of transcriptional repression regulate the level of endogenous BCAA biosynthesis in response to specific BCAA availability. We identify a *trans-*acting mechanism involving isoleucine-dependent repression by the global transcriptional regulator CodY and a *cis*-acting leucine-responsive attenuator, uncovering how *S*. *aureus* regulates endogenous biosynthesis in response to exogenous BCAA availability. Moreover, given that isoleucine can dominate CodY-dependent regulation of BCAA biosynthesis, and that CodY is a global regulator of metabolism and virulence in *S*. *aureus*, we extend the importance of isoleucine availability for CodY-dependent regulation of other metabolic and virulence genes. These data resolve the previous conflicting observations regarding BCAA biosynthesis, and reveal the environmental signals that not only induce BCAA biosynthesis, but that could also have broader consequences on *S*. *aureus* environmental adaptation and virulence via CodY.

## Author summary

To infect its human host, the bacterial pathogen *Staphylococcus aureus* must either take up nutrients from the surrounding environment or produce them itself. Previous studies have reported that *S*. *aureus* does not produce the amino acids leucine and valine, despite it possessing the genes to do so. In this study, we show that *S*. *aureus* does indeed produce leucine and valine, but only under certain nutritional conditions. We select for mutants of *S*. *aureus* able to grow in valine-depleted environments to uncover genetic variants that enable valine production. We discover genetic variants in a repressor protein and a region of non-coding DNA that both, when properly functioning, inhibit production of leucine and valine under nutrient-rich conditions. We further identify the nutritional conditions where the inhibition of leucine and valine production is relieved, thus revealing a previously overlooked role for another amino acid, isoleucine, in influencing nutrient metabolism. We show that isoleucine levels also influence expression of genes involved in the ability of *S*. *aureus* to cause disease. These findings help to reconcile conflicting reports regarding leucine and valine production in *S*. *aureus* and reveal nutritional cues that could influence its ability to cause infection.

## Introduction

*Staphylococcus aureus* is a serious human pathogen capable of causing infections that range from mild skin and soft tissue infections, to severe infections of the bone, muscle, heart and lung [1–4]. To survive and thrive in such diverse host environments, *S*. *aureus* must maintain sufficient levels of metabolites and co-factors to support virulence determinant production and replication [5,6]. The branched-chain amino acids (BCAAs; Ile, Leu, Val) represent an important group of nutrients for *S*. *aureus* metabolism and virulence, as they are required for synthesis of proteins and membrane branched-chain fatty acids (BCFAs), which are important for *S*. *aureus* membrane homeostasis and environmental adaptation. In addition to their nutritional importance, the BCAAs are key regulatory molecules in low GC-content Gram-positive bacteria, as they are activators of the global transcriptional regulator CodY. CodY coordinates expression of nutrient scavenging and synthesis systems, as well as virulence genes, upon depletion of both BCAAs and GTP [7–13]. The requirement of BCAAs for both *S*. *aureus* replication and niche adaptation necessitates that it either synthesize these nutrients or acquire them from the environment. Indeed, both BCAA biosynthesis [14–18] and transport [19–22] have been linked to promoting the virulence of other important pathogens in host environments.

Bacteria acquire BCAAs via dedicated active transporters, including BrnQ (Gram-negative and–positive bacteria), BcaP (Gram-positive bacteria), and the high affinity ATP-Binding Cassette (ABC) transporter LIV-I (Gram-negative bacteria) [23–36]. *S*. *aureus* encodes three BrnQ homologs (BrnQ1, BrnQ2, BrnQ3), and BcaP. BrnQ1 and BcaP transport all three BCAAs, with BrnQ1 playing a predominant role, and BrnQ2 is an Ile-dedicated transporter [24,25]. No appreciable BCAA transport function is associated with BrnQ3 [24]. Despite encoding the BCAA biosynthetic operon, *S*. *aureus* relies on the acquisition of BCAAs, most importantly Leu and Val, for rapid growth in media with excess or limiting concentrations of BCAAs, indicating that BCAA biosynthesis is typically repressed [24,25]. Paradoxically, biosynthesis remains repressed even in the absence of an exogenous source of Leu or Val, with growth of *S*. *aureus* observed only after a prolonged period, likely explaining why previous studies have been misled to conclude that *S*. *aureus* is auxotrophic for Leu and Val [37,38]. The molecular explanation for this phenotype in *S*. *aureus* has remained elusive.

Both Gram-positive and Gram-negative bacteria repress BCAA biosynthesis when intracellular levels are sufficient to support growth. In the Gram-negative bacteria *Escherichia coli* and *Salmonella enterica* sv. Typhimurium, this is regulated by transcriptional attenuation, which couples translation of a BCAA-rich peptide upstream of the biosynthetic genes with transcriptional termination, such that high levels of BCAAs prevent transcription of the biosynthetic genes [39–44]. In Gram-positive bacteria, including *Bacillus subtilis*, *Listeria monocytogenes*, and *S*. *aureus*, CodY represses transcription of the biosynthetic genes by binding to a CodY box and inhibiting binding of RNA polymerase [8,9,45–49]. Additional levels of regulation of the *ilv-leu* operon in the Gram-positive bacterium *B*. *subtilis* include activation by CcpA in response to glucose and repression by TnrA in response to nitrogen levels [50]. Additional fine-turning of the operon in this species is mediated by a Leu-responsive T-box riboswitch [50–53], as well as mRNA processing [54].

**Fig 1:**
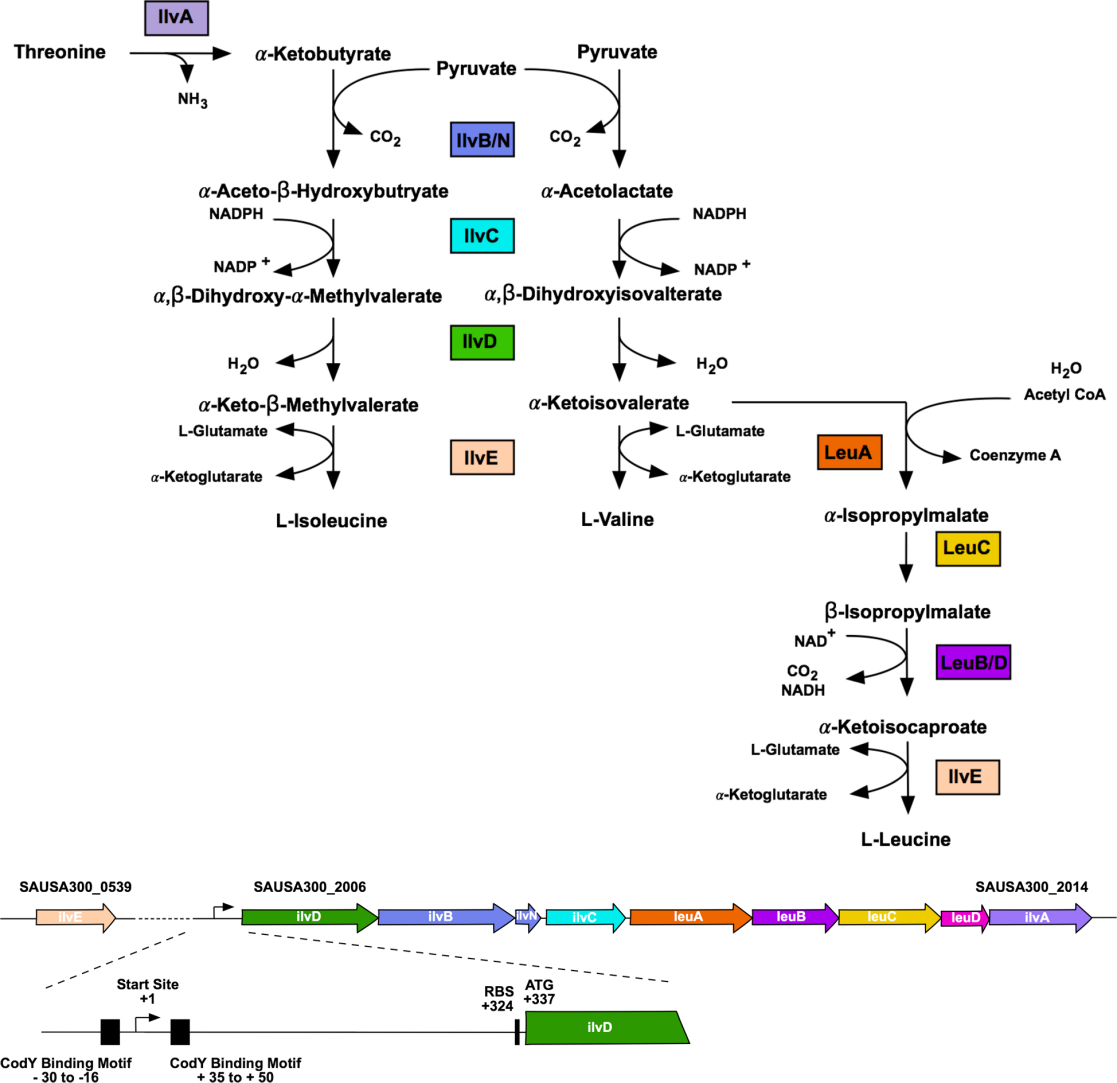
Organization of the *ilv*-*leu* operon in *S*. *aureus*. Top diagram is a schematic of the BCAA biosynthetic pathway in *S*. *aureus*. Bottom diagram depicts the genomic context of the BCAA biosynthetic genes in the USA300 FPR3757 genome. Regulatory features and their coordinates relative to the transcription start site are depicted, including the canonical CodY binding motifs [57,97] and the ribosome binding site (RBS).

The BCAA biosynthetic genes in *S*. *aureus*, encoded by the *ilvDBNCleuABCDilvA* operon (*ilv-leu*), and *ilvE* are similarly repressed by CodY; this regulator binds to two regions upstream of *ilvD* proximal to the transcriptional start site and two regions within the operon, proximal to *ilvC* and *leuC* (**Fig 1**) [7–9]. Repression is also mediated by the essential genes *gcp* and *yeaZ* through an unknown mechanism [55,56]. Given that CodY transcriptional repression should be alleviated in the absence of BCAAs, it is unclear why in the case of Leu and Val specifically, growth remains inhibited when either of these two amino acids is absent from the growth medium. We therefore investigated the mechanisms governing these phenotypes in *S*. *aureus* to resolve this paradox and to identify the signals required to induce synthesis. Here, we unravel the complex regulation of BCAA biosynthesis in *S*. *aureus*, by demonstrating that control is mediated by both *trans* and *cis* acting mechanisms of repression. We identify the metabolic cues regulating each mechanism, therefore revealing how *S*. *aureus* controls its preference for exogenous BCAAs, and the conditions under which endogenous synthesis is induced. In doing so, we uncover an unappreciated role for Ile in CodY-dependent regulation, demonstrating that it is this BCAA that plays a dominant role in controlling the expression of genes involved in BCAA synthesis and transport. Moreover, since we show that Ile can dominate CodY-dependent gene expression, we highlight an important role for Ile limitation in virulence gene expression, where the absence of this BCAA can induce expression of nuclease, a known CodY-dependent virulence factor.

## Results

### Growth of *S*. *aureus* in response to BCAA deprivation

**Fig 2:**
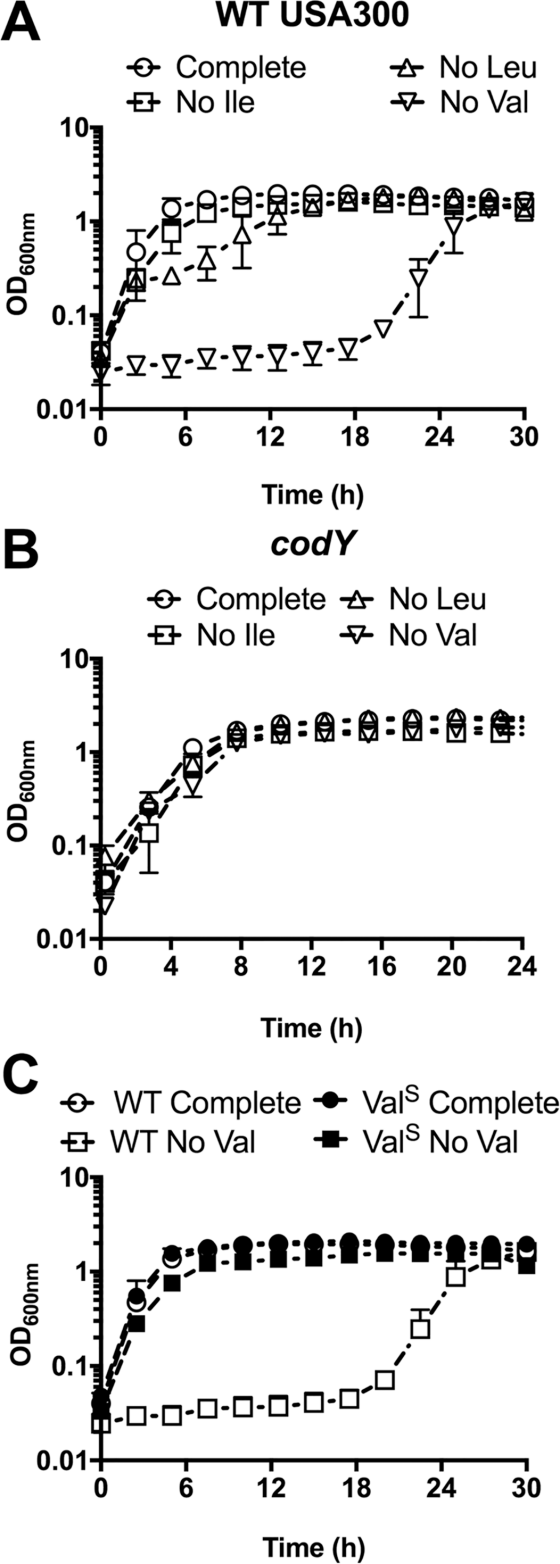
Growth of *S*. *aureus* upon BCAA depletion. A) WT USA300 was pre-grown in complete CDM to mid-exponential phase and then sub-cultured into either complete CDM or CDM with BCAAs omitted, as indicated. B) USA300 with a transposon insertion in *codY* (*codY*::ϕNΣ) was pre-grown in complete CDM to mid-exponential phase, and then sub-cultured into either complete CDM or CDM with BCAAs omitted, as indicated. C) Cells recovered from the CDM with no Val in panel A were plated. A single colony was selected (Val^Sup^ (abbreviated Val^S^) —filled symbols) and subjected to growth in complete CDM and CDM with no Val. Growth was compared to the parental WT strain (open symbols) in the same conditions. Data are the mean +/- SD of three biological replicates.

*S*. *aureus* has previously been reported as auxotrophic for Leu and Val [37,38], despite possessing a complete BCAA biosynthetic operon. In contrast to these reports, we have observed that *S*. *aureus* is indeed able to grow in the absence of Leu and Val following an extended growth period [24], which might in part explain the discrepancy in these observations. Curiously, when investigating the kinetics of *S*. *aureus* growth in response to deprivation of each individual BCAA, we found differing growth phenotypes, even though all enzymes required for the synthesis of each of the BCAAs are encoded from the same biosynthetic loci. For example, when grown in chemically-defined media (CDM) lacking Leu, *S*. *aureus* exhibited a growth lag of 6–8 h, and when grown in CDM lacking Val *S*. *aureus* exhibited a growth lag of ~ 20 h, relative to its growth in complete CDM (**Fig 2A**). In contrast, growth of *S*. *aureus* in CDM lacking Ile was comparable to growth in complete CDM (**Fig 2A**). These observations were particularly perplexing given the known mechanism, via CodY, regulating BCAA biosynthesis. CodY represses the BCAA biosynthetic operon, such that inactivation of *codY* results in growth of *S*. *aureus* in media lacking either Ile, Leu, or Val (**Fig 2B**). Given that all three BCAAs have been reported to individually activate CodY DNA binding activity *in vitro* [13,57,58], it was surprising to observe the differences in growth upon omission of the individual BCAAs from the growth medium, and how different it was from that of WT *S*. *aureus* and a *codY* mutant (compare panels A and B in Fig 2). We therefore hypothesized that the individual BCAAs differentially regulate CodY activity during growth, and that at least one additional mechanism regulates BCAA biosynthesis.

To uncover the molecular mechanisms governing these phenotypes, we first questioned whether the absence of Leu or Val selects for mutations that enable growth in the absence of these BCAAs. To address this question, we recovered cells that had grown up following the growth lag in media lacking Leu (CDM^-Leu^) or Val (CDM^-Val^), and then sub-cultured these isolates back into the same medium from which they were recovered. Cells recovered from CDM^-Leu^ medium exhibited the same growth delay upon sub-culture into the same medium, indicating that this condition does not select for mutations. Conversely, cells recovered from CDM^-Val^ medium grew readily when re-inoculated into CDM^-Val^, suggesting that they were synthesizing Val (-Val suppressors, referred to as Val^Sup^) (**Fig 2C**). These results suggest that growth in the absence of exogenous Leu requires a regulatory adaptation, whereas growth in the absence of Val selects for a heritable mutation. We hypothesized that identification of the genetic mutations permitting growth of *S*. *aureus* in the absence of exogenous Val would reveal important regulators of the BCAA biosynthetic operon and, in-turn, would help reveal the mechanisms behind the BCAA-specific growth phenotypes.

### Growth in media lacking Val selects for mutations in *codY*

**Fig 3:**
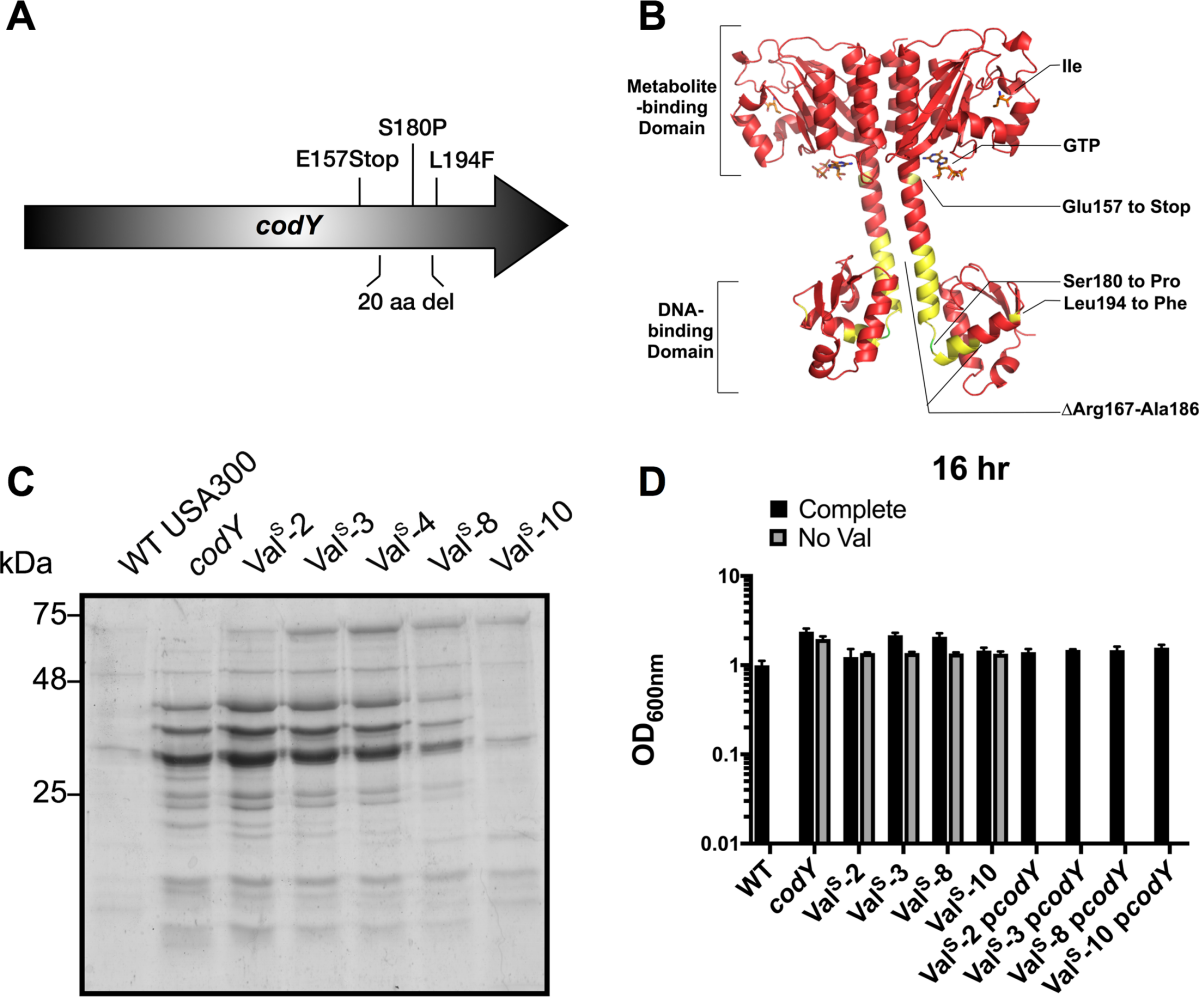
The absence of exogenous valine selects for mutations that inactivate CodY. A) Schematic representation of the mutations identified in CodY. B) Mutations identified are indicated on the CodY structure (PDB ID:5EY0) in yellow, except for the Ser180 to Pro mutation, which is indicated in green. CodY ligands, Ile and GTP, are coloured based on atomic composition. C) Strains were pre-grown in TSB to mid-exponential phase, then sub-cultured into TSB for 16 hr. Supernatants were collected and proteins were precipitated using TCA. Protein samples were normalized to the equivalent of 5 ODs and run on a 12% SDS-PAGE gel. D) Strains with unique mutations in *codY* (Val^Sup^-2 carries an identical mutation to Val^Sup^-4) were pre-grown in complete CDM to mid-exponential phase, then sub-cultured into either complete CDM or CDM with Val omitted. OD_600nm_ was read after 16 hr of growth. USA300 with a transposon insertion in *codY* (*codY*::ϕNΣ) was used for comparison. Val^Sup^ is abbreviated to Val^S^. Data are the mean +/- SD of three biological replicates.

Since a *codY* mutant synthesizes BCAAs and is, thus, capable of growth in the absence of BCAAs, we reasoned that the absence of exogenous Val may select for mutations in *codY*. We again grew cells in the absence of Val, isolated mutants from twelve independent cultures (Val^Sup^ mutants) and amplified the *codY* gene by PCR. Five out of the twelve mutants contained mutations in *codY*; one had a point mutation resulting in a premature stop codon, two had a 60-bp deletion, and two had independent point mutations resulting in nonsynonymous mutations (**Table 1**) and (**Fig 3A**). We then mapped the mutations to identify their position within the CodY protein structure (PDB ID:5EY0) [59]. All mutations occurred in the linker region between the metabolite sensing domain and the DNA-binding domain (**Fig 3B**). We used secreted protein profiles as a read-out of CodY function, since CodY represses many secreted proteins [60] and therefore the secreted protein profile of a *codY* mutant differs substantially from WT. The secreted protein profiles of the Val^Sup^ mutants with confirmed mutations in *codY* resembled the protein profile of the *codY* mutant, except for Val^Sup^-10 mutant (**Fig 3C**), indicating that all but one of our *codY* mutations result in an inactive CodY protein, at least insofar as its ability to repress synthesis of secreted proteins. The growth phenotype for each of the unique Val^Sup^ strains with confirmed mutations in *codY* could be reverted to WT-like growth in CDM^-Val^ through complementation with an intact copy of the *codY* gene *in trans* (**Fig 3D**). These results confirm that *codY* inhibits Val synthesis, and that all of the Val^Sup^ *codY* mutants, along with the *codY* insertion mutant (*codY*::ϕNΣ), alleviate this inhibition.

**Table 1:** Mutations identified in CodY.

Mutants	Position^a^	Genetic Mutation	Protein Mutation
Val^Sup^-2	1260149–1260208	60 bp deletion	ΔArg_167_-Ala_186_
Val^Sup^-3	1260119	G to T	Glu_157_ to Stop
Val^Sup^-4	1260149–1260208	60 bp deletion	ΔArg_167_-Ala_186_
Val^Sup^-8	1260188	T to C	Ser_180_ to Pro
Val^Sup^-10	1260230	C to T	Leu_194_ to Phe

^a^Position in the USA300 FPR3757 genome (NC_007793.1)

### Growth in media lacking Val selects for mutations in the 5’UTR of *ilvD*

**Fig 4:**
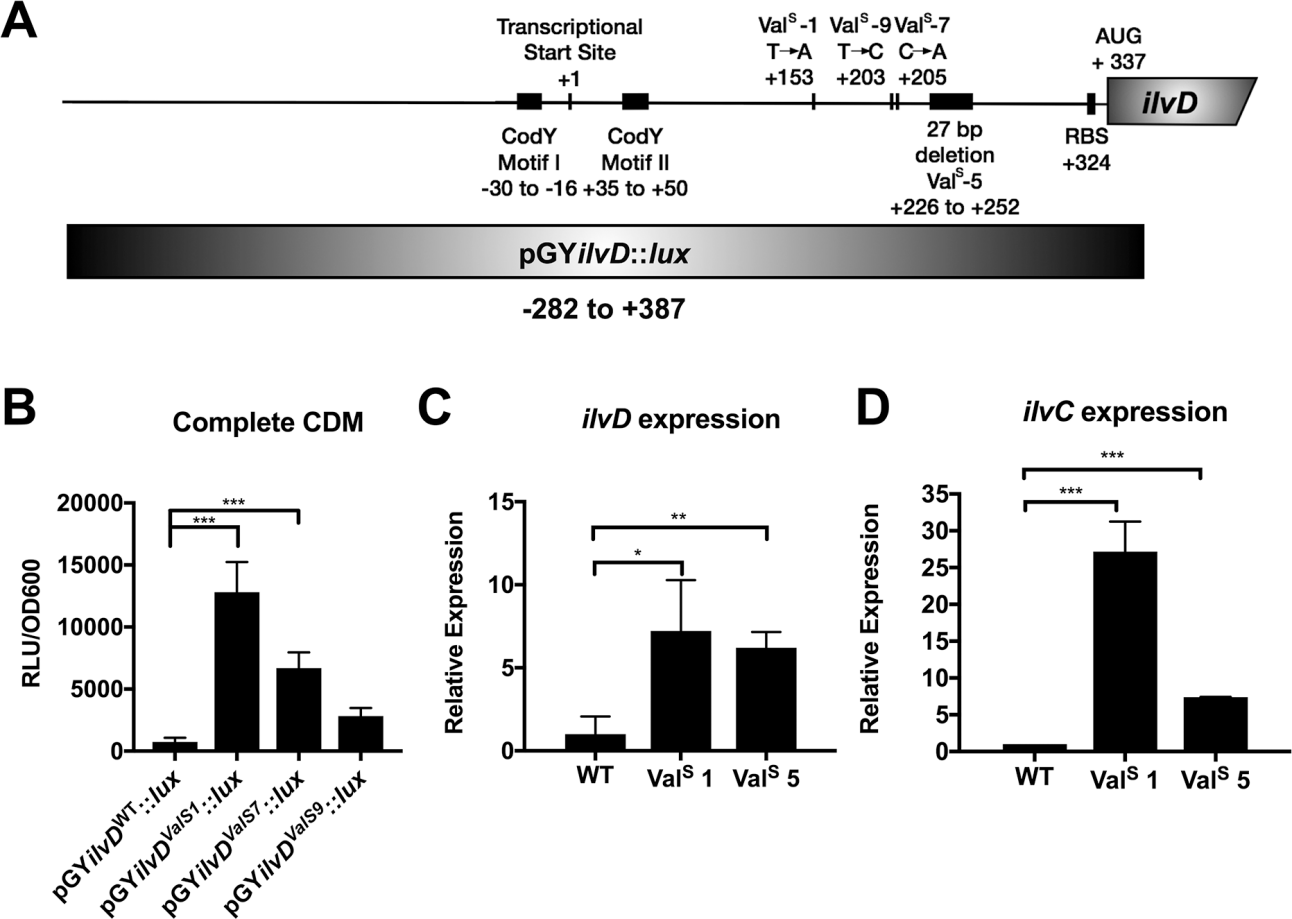
Mutations in the *ilvD* promoter result in an increase in promoter activity and *ilv-leu* operon expression. A) Position map of the mutations identified in the *ilvD* promoter relative to the transcriptional start site, the canonical CodY binding motifs, and the region used for cloning in the promoter:reporter construct. B) WT USA300 with the pGY::*lux* plasmid containing either the WT *ilvD* promoter, or the mutant *ilvD* promoter was grown in complete CDM to mid-exponential phase and then sub-cultured into complete CDM. Luminescence was read at mid-exponential phase and normalized to the OD_600nm_. Data are the mean +/- SD of three biological replicates. Data were analyzed by one-way ANOVA with Dunnet’s multiple comparison test. *** *P* < 0.001. C, D) Strains were grown in complete CDM to mid-exponential phase and then sub-cultured into complete CDM. Cells were harvested at mid-exponential phase and RNA was isolated. Expression of *ilvD* and *ilvC* was normalized to expression of *rpoB*. Val^Sup^ is abbreviated to Val^S^. Data are the mean +/- SD of three biological replicates. Data were analyzed by an Student’s unpaired *t*-test. *** *P* < 0.001, ** *P* < 0.01, * *P* < 0.05.

The remaining seven Val^Sup^ mutants that did not have mutations in the *codY* gene may have acquired other mutations that indirectly affect the ability of CodY to regulate the *ilv-leu* operon (e.g. mutations in GTP synthesis). To address this possibility, we assessed the secreted protein profiles of these mutants and all seven were found to exhibit profiles comparable to the WT strain (**S1 Fig**). These results suggest the mutations occurring within these Val^Sup^ strains affect a CodY-independent mechanism of BCAA synthesis regulation. We therefore performed whole genome sequencing to identify the nature of these mutations. This revealed that all seven of these Val^Sup^ strains had mutations in the 5’ untranslated region (UTR) upstream of *ilvD*, with a total of three unique point mutations and one 27-bp deletion (**Table 2**). The mutations did not overlap with the known promoter features upstream of the *ilvD* gene (i.e. the CodY binding motifs) (**Fig 4A**). To confirm that mutations in the 5’UTR of *ilvD* result in an increase in expression of the *ilv*-*leu* operon, which would yield the phenotype of growth in CDM^-Val^ without delay (Fig 2, panel C), we generated a luminescence reporter of the *ilvD* promoter by cloning the 5’UTR of *ilvD* into the pGY::*lux* vector (**Fig 4A**). Within this reporter construct, we then mutated the 5’UTR to contain the three point mutations identified from genome sequencing of the mutants (Val^Sup^-1, Val^Sup^-7, and Val^Sup^-9). Two of the mutant sequences (Val^Sup^-1 and Val^Sup^-7) resulted in a statistically significant increase in *ilvD* promoter activity, and the third mutant sequence (Val^Sup^-9) resulted in a trend towards increased promoter activity, although not significant (**Fig 4B**). Furthermore, using qPCR, we observed that levels of *ilvD* and *ilvC* transcripts were elevated when we examined two of the mutants compared to the WT strain (**Fig 4C and 4D**). Together, these data suggest that the mutations in the 5’UTR of *ilvD* relieve repression of the *ilv*-*leu* operon.

**Table 2:** Mutations identified in the 5’ UTR of *ilvD*.

Mutants	Position^a^	Position relative to *ilvD* transcriptional start site	Mutation
Val^Sup^-1	2164689	+153	T to A
Val^Sup^-5	2164762–2164788	+226 to +252	27 bp deletion
Val^Sup^-6	2164762–2164788	+226 to +252	27 bp deletion
Val^Sup^-7	2164741	+205	C to A
Val^Sup^-9	2164739	+203	T to C
Val^Sup^-11	2164762–2164788	+226 to +252	27 bp deletion
Val^Sup^-12	2164739	+203	T to C

^a^Position in the USA300 FPR3757 genome (NC_007793.1)

### Identification of a putative attenuator and terminator

**Fig 5:**
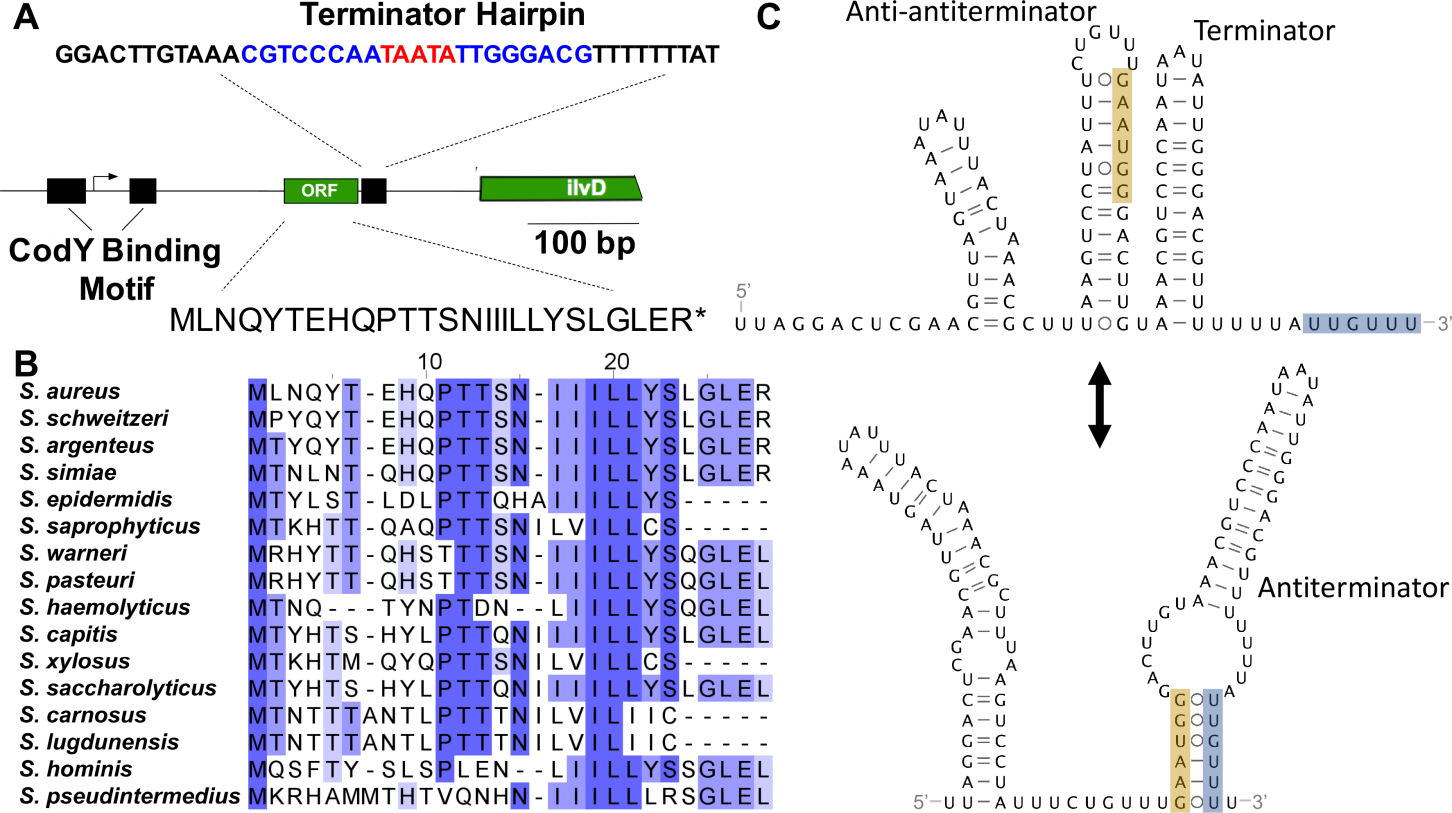
Attenuator features upstream of *ilvD*. A) The position and sequence of the terminator hairpin is shown for the WT USA300 strain and the Val^Sup^-5 mutant strain. The terminator sequence is in bold, with the stem highlighted in blue and the loop highlighted in red, followed by a poly-U tail. The putative open reading frame (ORF) and the corresponding translated attenuator peptide is shown for the WT USA300 strain and the Val^Sup^-1 mutant. The positions of the features are indicated relative to the transcriptional start site identified in Majerczyk *et al*., 2010. B) Multiple sequence alignments of the homologous peptide found in the *ilvD* leader from other staphylococcal species. C) Alternate RNA secondary structures of a terminator and antiterminator predicted in the *ilvD* leader using the Mfold webserver. The alternatively paired segments in the antiterminator stem are highlighted in orange and gray. Base pairing is shown using Leontis-Westhof notation. Val^Sup^ is abbreviated to Val^S^.

We next investigated whether the 5’UTR of *ilvD* contained a *cis*-regulatory element, initially considering a T-box riboswitch, since the *ilv-leu* operon in *B*. *subtilis* is regulated by a tRNA^Leu^-responsive T-box riboswitch [51,53]. Predictive structure analysis and sequence comparison of the *ilvD* 5’UTR to known T-box riboswitch sequences revealed that although the *S*. *aureus ilvD* 5’UTR contains some features that loosely resemble T-box riboswitches (**S2 Fig**), it lacks the conserved Stem 1 motifs and structures essential to tRNA anchoring and decoding [61]. We next considered translation-dependent transcriptional regulation (i.e. attenuation), since BCAA-rich leader peptides have been found to regulate BCAA biosynthetic genes in *E*. *coli* [42] and *S*. *typhimurium* [43,44], and are predicted to regulate BCAA synthesis in *Lactococcus lactis* sp. *lactis* [62], *Corynebacterium glutamicum* [63,64], and *Streptococcus* spp. [65]. A search for open reading frames (ORFs) in the *ilvD* leader sequence revealed a short coding region that would be predicted to encode a 26-aa peptide. The predicted peptide contains a string of three Ile codons followed by two Leu codons, and an additional three interspersed Leu codons (**Fig 5A**). A putative ribosome binding site was also identified 9 nucleotides (nts) upstream of the start codon and a putative terminator hairpin structure is located 52 nts downstream from the peptide stop codon, consistent with transcription termination (**S3 Fig**). We found that the Ile and Leu codons in the peptide were highly conserved across the staphylococci (**Fig 5B**), as was the predicted terminator stem loop structure (**Fig 5C and S3 Fig**), suggesting that these features are biologically relevant. Secondary structure predictions revealed an alternative mRNA structure could also form that sequesters the terminator poly-U within an antiterminator (**Fig 5C**). In the antiterminator fold, the terminator stem-loop is intact, but two upstream stem-loops refold into a new, long stem-loop shifted further upstream. This rearrangement frees a 5’-GAAUGG-3’ motif to pair with 5’-UUGUUU-3’ in the terminator poly-U tail (**Fig 5C**). Ribosome pausing within these regions could foreseeably disrupt folding to favor antiterminator formation and drive transcription of the *ilv-leu* operon.

When we considered how the mutations that were selected for in media lacking Val might disrupt these features, we found that the 27-bp deletion in Val^Sup^-5/6/11 deletes the predicted terminator stem-loop and the T to A mutation in Val^Sup^-1 changes a Leu codon in the leader peptide to a stop codon (**Fig 5A**). These mutations would therefore be predicted to relieve repression of transcription, supporting that these are biologically relevant features. The T to C mutation in Val^Sup^-9/12 and the C to A mutation in Val^Sup^-7 occur in predicted secondary structural elements that stabilize terminator formation, and could also relieve repression. We therefore hypothesize that expression of the BCAA biosynthesis operon is regulated by Leu-dependent attenuation in *S*. *aureus*. We predict that, in conditions of high Leu availability, translation of the attenuator peptide promotes formation of the terminator hairpin and subsequently transcriptional termination. In conditions of low Leu availability, the ribosome stalls during translation of the attenuator peptide, promoting formation of the antiterminator hairpin and leading to transcriptional read-through. This region, upstream of *ilvD*, is henceforth referred to as the attenuator sequence and not the 5’UTR.

### Ile and Leu regulate the *ilv* operon via *trans*- and *cis*-acting mechanisms, respectively

**Fig 6:**
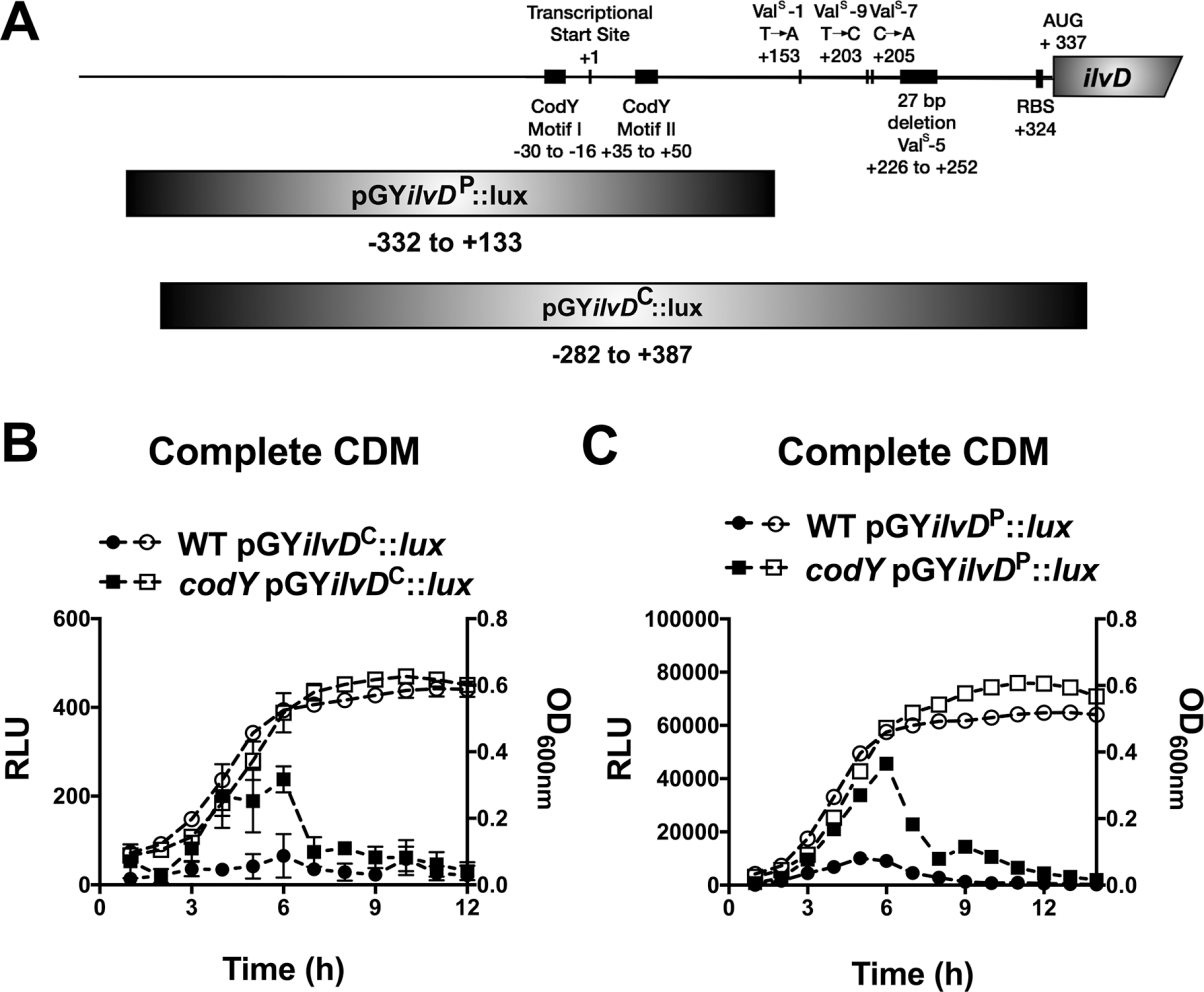
Characterization of *cis* and *trans* regulation of *ilvD* expression. A) Diagram of the regions cloned into the pGY::*lux* vector to create pGY*ilvD*^P^::*lux* and pGY*ilvD*^C^::*lux*. B,C) Strains were pre-grown in complete CDM to mid-exponential phase and then sub-cultured into complete CDM in a 96-well plate. Luminescence (left axis, filled shapes) and OD_600nm_ (right axis, open shapes) were read hourly. Val^Sup^ is abbreviated to Val^S^. Data are the mean +/- SD of three biological replicates.

The *in vitro* selection experiment revealed two mechanisms involved in repression of the *ilv*-*leu* operon, and yet the growth data (see **Fig 2A**) suggest that the operon is fully repressed only under conditions of Val deprivation and not Ile or Leu deprivation. We therefore continued to investigate how these mechanisms respond to deprivation of the individual BCAAs to explain these unique growth phenotypes. To test our hypothesis that Leu availability regulates expression of the *ilv*-*leu* operon via attenuation, we used our luminescence reporter construct containing the attenuator sequence cloned into the pGY::*lux* vector to examine how it responds to Leu deprivation. We were also interested to investigate how BCAA availability regulates CodY regulation of the *ilv*-*leu* operon, since the growth phenotypes of *S*. *aureus* in the absence of Leu or Val suggests that depletion of these nutrients alone is not sufficient to relieve CodY-dependent repression (compare **Fig 2A and 2B**). To study CodY-dependent promoter activity in isolation of attenuation, we generated a second reporter construct (partial promoter; pGY*ilvD*^*P*^::*lux*) that lacked the attenuator sequence and contained only the CodY binding sequence and compared this to the original construct (complete promoter; pGY*ilvD*^*C*^::*lux*) that contained both regulatory elements (**Fig 6A**). We first confirmed that both constructs responded to CodY and, indeed, observed higher promoter activity in the *codY* mutant compared to the WT strain that peaked during mid-exponential growth (**Fig 6B and 6C**). All endpoint pGY::*lux* experiments from this point on are therefore the luminescence normalized to the optical density of mid-exponential phase cells (RLU/OD_600_). We note that, generally, the partial promoter fusion has higher activity than the complete promoter fusion, likely due to omitting the attenuator sequence.

**Fig 7:**
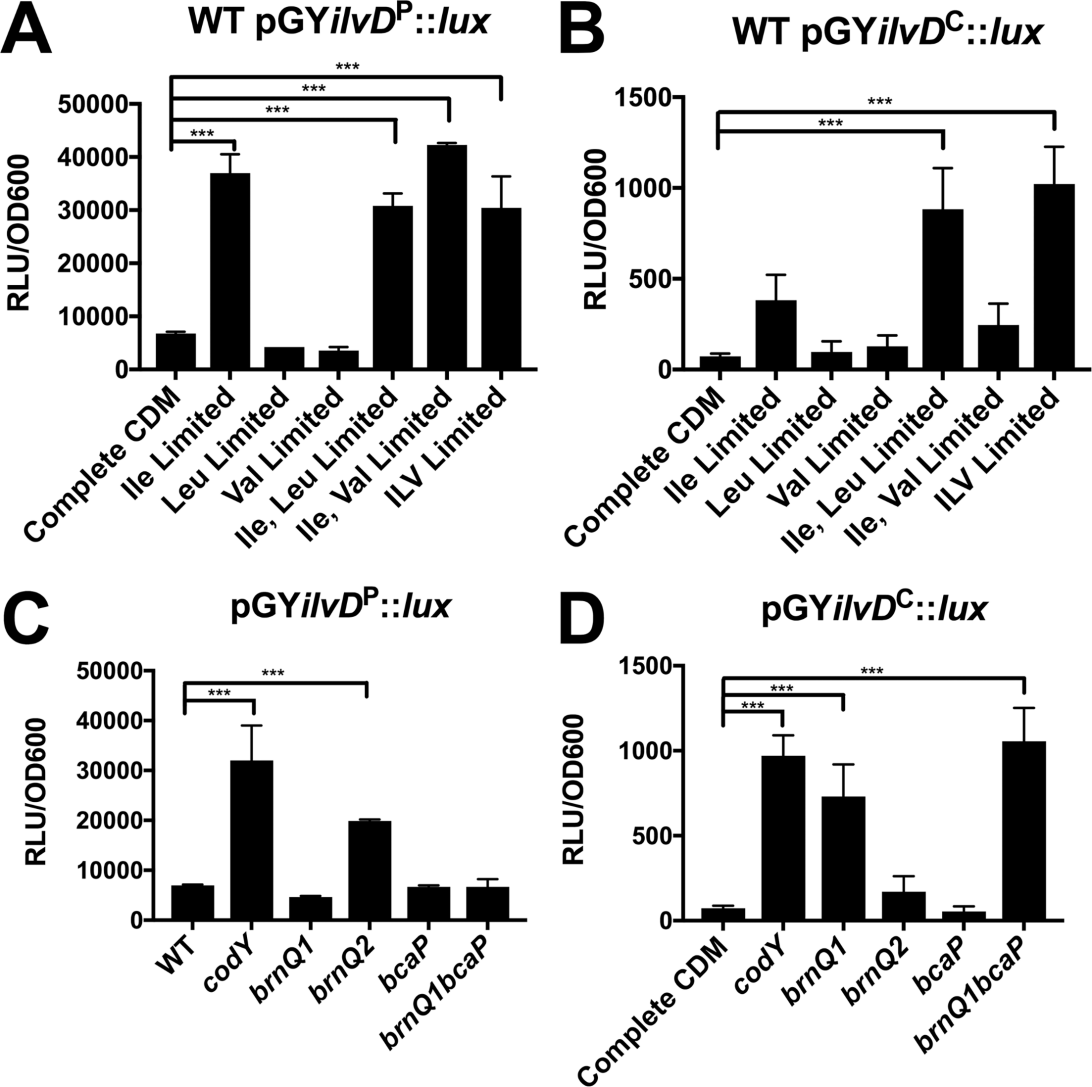
Ile is the predominant BCAA to regulate CodY activity on the *ilvD* promoter. WT *S*. *aureus* containing the *lux* reporter vector with either A) the partial *ilvD* promoter region (pGY*ilvD*^P^::*lux*) or B) the complete *ilvD* promoter region (pGY*ilvD*^C^::*lux*) was pre-grown in complete CDM to mid-exponential phase and then sub-cultured into either complete CDM or CDM with limiting concentrations of BCAAs, as indicated. Concentrations of Ile, Leu, and Val in complete CDM are 228 μM, 684 μM, 684 μM, respectively. Concentrations of Ile, Leu, and Val in limited media are 23 μM, 68 μM, and 68 μM, respectively. Luminescence values were read when cells reached mid-exponential phase and were normalized to the OD_600nm_. Data are the mean of three biological replicates +/- SD. *S*. *aureus* strains with mutations in either *codY* (*codY*::ϕNΣ) or BCAA transporters and containing either C) the partial *ilvD* promoter region (pGY*ilvD*^P^::*lux*) or D) the complete *ilvD* promoter region (pGY*ilvD*^C^::*lux*) were pre-grown in complete CDM to mid-exponential phase and then sub-cultured into complete CDM. Luminescence values were read when cells reached mid-exponential phase and were normalized to the OD_600nm_. Data are the mean of three biological replicates +/- SD. Data were analyzed by one-way ANOVA with Dunnet’s multiple comparisons test. *** *P* < 0.001.

We next assessed promoter activity in response to depletion of each BCAA. Since complete omission of Leu and Val from the growth medium significantly attenuates *S*. *aureus* growth, we instead limited their concentrations to 10% of that in complete CDM to minimize differences in growth. We first examined CodY-dependent promoter activity using the pGY*ilvD*^*P*^::*lux* construct. Promoter activity increased to levels comparable to the *codY* mutant only upon Ile limitation, and limitation of Leu or Val in combination with Ile did not alter *ilvD* promoter activity any further (**Fig 7A**), indicating a predominant role of Ile in regulating CodY activity on the *ilvD* promoter. We next examined the effect of BCAA limitation on attenuator-dependent regulation. *ilvD* promoter activity of the pGY*ilvD*^*C*^::*lux* construct also increased upon Ile limitation, however, we also observed a further increase in promoter activity when Leu and Ile were limited simultaneously (**Fig 7B**). These data suggest that the attenuator sequence, which is unique to the pGY*ilvD*^*C*^::*lux* construct, responds to Leu availability. This is consistent with our hypothesis that the attenuator represses the BCAA operon in response to Leu.

We previously identified mechanisms of BCAA transport in *S*. *aureus*, including BrnQ1 and BcaP, which transport Ile, Leu and Val, and BrnQ2, a dedicated Ile transporter [24,25]. To determine the contribution of each of these transporters to either CodY-dependent or attenuator-dependent regulation of BCAA biosynthesis, we assessed *ilvD* promoter activity in various BCAA transporter mutants. *ilvD* promoter activity of the pGY*ilvD*^P^::*lux* construct increased only in the *brnQ2* mutant (**Fig 7C**), whereas the pGY*ilvD*^C^::*lux* increased in the *brnQ1* and *brnQ1bcaP* mutants (**Fig 7D**). These data indicate that BrnQ2-dependent Ile transport is linked to CodY activity and BrnQ1/BcaP-dependent Leu transport is linked to attenuation. Notably, using the complete *ilvD* promoter region, we did not observe a change in promoter activity in the *brnQ2* mutant. We have previously shown that *brnQ1* is upregulated in a *brnQ2* mutant and consequently a *brnQ2* mutant takes up more Leu and Val permitting enhanced growth in media limited for these BCAAs [24]. We thus postulate that in a *brnQ2* mutant, the increased Leu uptake causes repression of *ilvD* promoter activity via the attenuator and overrides the CodY-dependent Ile response.

### All three BCAAs activate CodY DNA-binding activity

**Fig 8:**
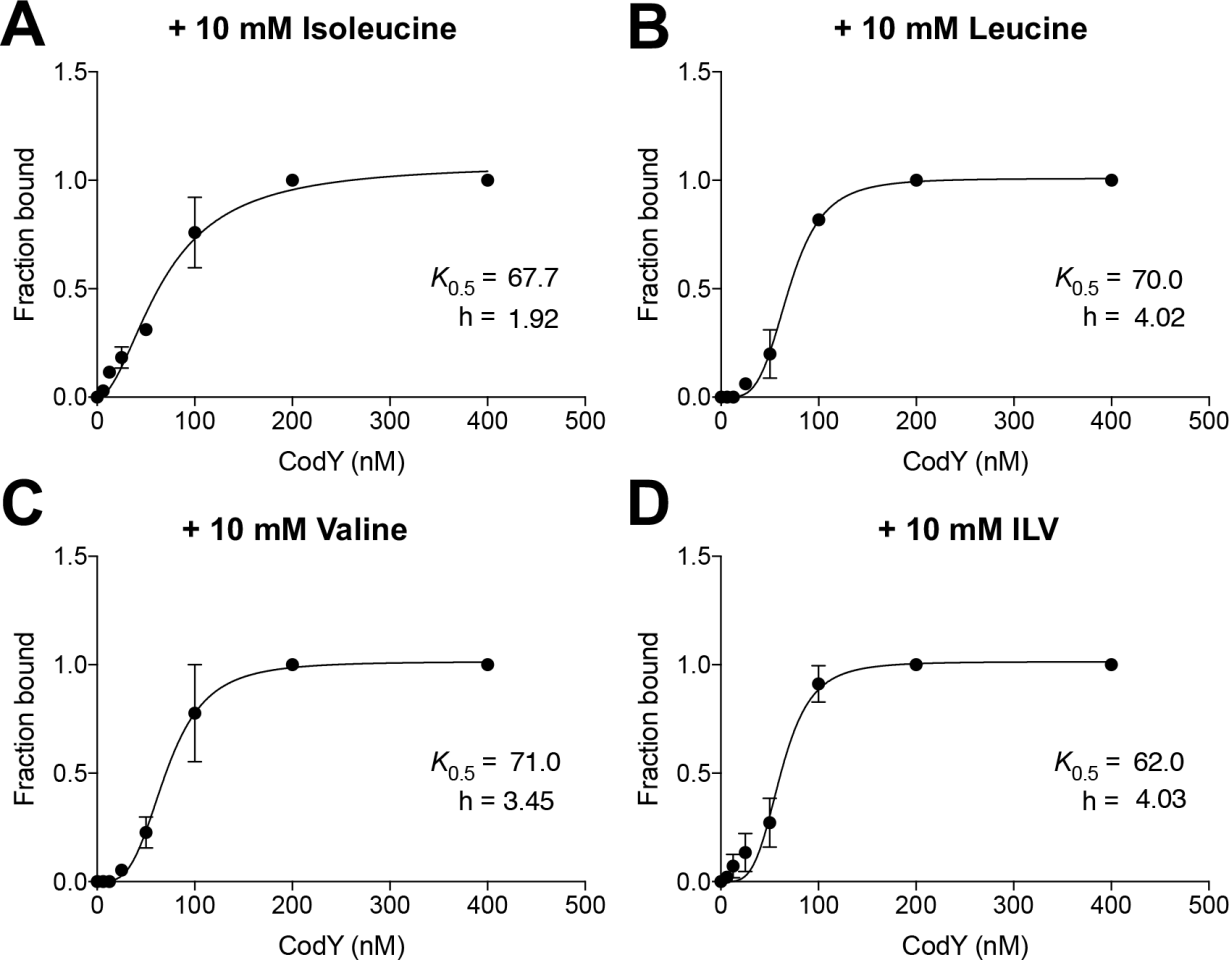
CodY binds all three branched-chain amino acids *in vitro*. Fraction of bound *ilvD*_*266*_*p*^*+*^ fragment was analyzed in EMSAs containing GTP and A) isoleucine, B) leucine, C) valine, or D) all three amino acids (ILV). The binding constants (*K*_0.5_) and Hill coefficients (h) were determined by fitting the data to the Hill equation [98]. Data points indicate the mean +/- SEM of two independent experiments. Error bars were plotted for all points; in some cases, the error bars are too small to see.

We next revisited the DNA binding activity of CodY at the *ilvD* promoter in the presence of each BCAA to compare CodY activity *in vitro* vs during growth. To test whether Ile activates CodY to bind DNA more efficiently than Leu or Val, we analyzed the interaction of CodY with a fluorescently-labeled DNA fragment (*ilvD*_*266*_*p*^*+*^) containing the annotated CodY regulatory region of *ilvD* [9]. Ile-activated CodY formed DNA:CodY complexes with as little as 6.5 nM CodY monomer, whereas Leu- and Val-activated CodY formed similar, multiple DNA:protein complexes as Ile-activated CodY, but required ~4-fold more CodY protein (**S4A–S4C Fig).** However, band densitometry analysis and fitting the data to a Hill equation revealed that the apparent binding constant values were essentially identical for all ligands tested (**Fig 8)**. We did not observe an additive effect of all three BCAAs on CodY binding activity (**S4D Fig)**. Thus, CodY binds all three amino acids *in vitro*.

### Absence of exogenous Ile restores growth in media lacking Leu, but not Val

**Fig 9:**
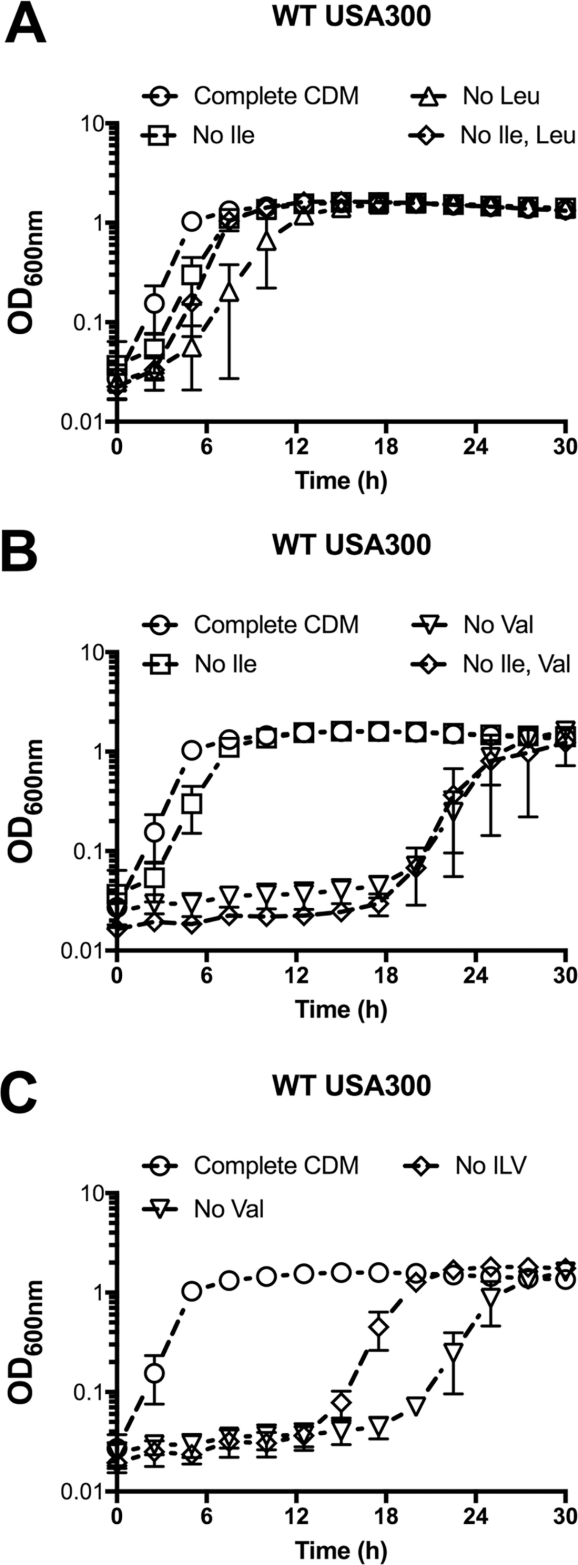
Omission of Ile from CDM restores growth in media lacking Leu, but not Val. A-C) WT USA300 was pre-grown in complete CDM to mid-exponential phase and then sub-cultured into either complete CDM or CDM with amino acids omitted, as indicated. Data are the mean +/- SD of three biological replicates.

Thus far, our data provide insight into the molecular mechanisms governing the BCAA-specific growth phenotypes of *S*. *aureus* observed in panel A of Fig 2. In complete CDM, the *ilv*-*leu* operon is repressed in an Ile-dependent manner via CodY and in a Leu-dependent manner via the attenuator peptide. Omission of Ile from the growth medium relieves CodY repression of the *ilv*-*leu* operon, resulting in Ile synthesis, which supports rapid growth in the absence of an exogenous Ile source. In media lacking Val, CodY remains active and the presence of Leu triggers transcriptional termination of the *ilv-leu* operon via the attenuator peptide; thus *S*. *aureus* is unable to synthesize Val and consequently unable to grow unless either of the aforementioned mechanisms is mutated. Omission of Leu from the growth medium relieves attenuator-dependent repression, however CodY remains active, and consequently, Leu synthesis is only partially relieved, resulting in a reduced growth rate. It therefore follows that simultaneous omission of Ile and Leu or Val should permit growth of *S*. *aureus* due to de-repression of CodY. Indeed, we found that the growth of *S*. *aureus* in CDM lacking Ile and Leu initiated more rapidly than growth in CDM lacking Leu alone, indicating that the reduced growth rate in CDM^–Leu^ is due to Ile-dependent CodY repression (**Fig 9A**). Unexpectedly, *S*. *aureus* grown in CDM lacking Ile and Val resembled growth of *S*. *aureus* in media lacking Val alone (**Fig 9B**). Since the presence of Leu also contributes to repression of the operon, we further examined growth of *S*. *aureus* in CDM lacking all three BCAAs, however the growth of *S*. *aureus* remained attenuated, with no observable growth until a prolonged period of ~ 16 hr (**Fig 9C**). These data are curious given that the immediate precursor of Val is also a precursor of Leu, and the aminotransferase (IlvE) that converts ketoisovalerate to Val also produces Ile and Leu (**Fig 1**). Since our promoter:reporter data demonstrate that the *ilvD* promoter is active in media limited for all three BCAAs (**Fig 7A and 7B**) and thus the operon is presumed derepressed, we postulate that the growth impairment in media lacking all three BCAAs is related to enzymatic activity of the biosynthetic enzymes, whereby either the aminotransferase exhibits substrate bias towards Ile or Leu synthesis, or there is negative or positive cross-regulation between the pathways. For example, the threonine deaminase (IlvA) required only for Ile synthesis (**Fig 1**) is activated by Val in *E*. *coli* and *B*. *subtilis* [66,67]. Therefore, it is possible that in the absence of Val, Ile synthesis is reduced, contributing to the growth impairment in media lacking both Ile and Val.

We were curious to investigate whether mutations in the promoter region of *ilvD* arise in the environment, reasoning that *S*. *aureus* might encounter Val-limited environments that impair growth and therefore select for mutations in the regulatory mechanisms involved in repression. We compared the nucleotide sequence of the *ilvD* promoter region from USA300 FPR3757 to all complete genome sequences of *S*. *aureus*. Overall, there was high sequence conservation, however, several variants were identified in the putative regulatory regions (**S5 Fig**). Intriguingly, two variants occur in the putative ORF upstream of *ilvD* and both alter the number of Leu codons in the peptide (located at +151 and +162 in **S5 Fig**). Several sequence variants also occur in the first CodY binding region. Ongoing studies will investigate the consequence of these mutations on the level *ilv*-*leu* expression and subsequent BCAA biosynthesis in these strains.

### Ile deprivation induces expression of a nutrient transporter and nuclease

Our data revealed an unexpected role for Ile, and not Leu or Val, in regulating CodY activity on the *ilvD* promoter. The predominant role for Ile could have important implications for *S*. *aureus* physiology and virulence given that CodY is considered a master regulator of metabolism and virulence gene expression in *S*. *aureus* [7–9,49,60]. It was therefore of high interest to us to investigate whether the predominant role of Ile in regulating CodY activity was unique to *ilvD* or if it extended to other CodY-regulated genes.

**Fig 10:**
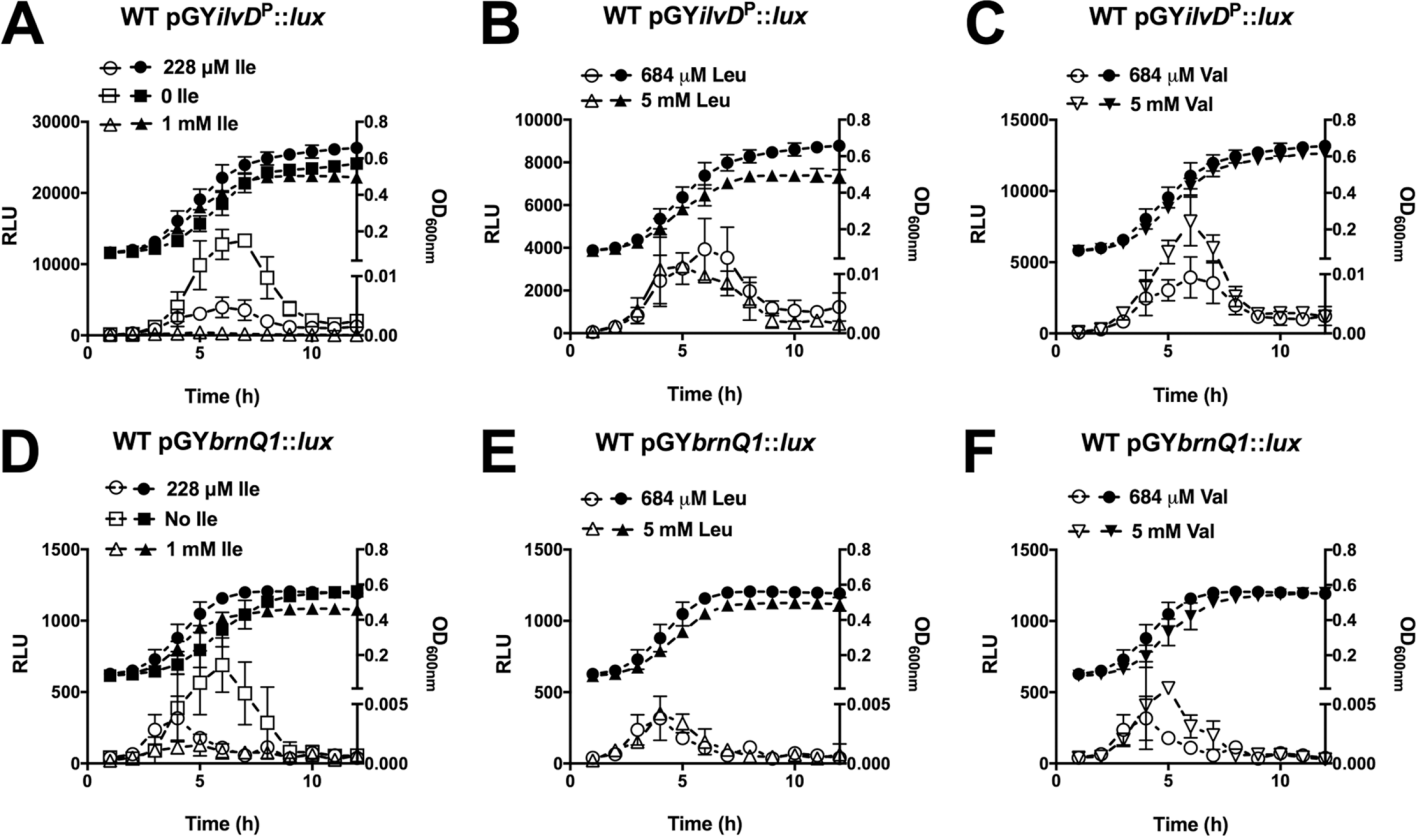
Ile is the predominant BCAA to regulate CodY activity on the *brnQ1* promoter. A-F) WT *S*. *aureus* containing the *lux* reporter vector with either A-C) the partial *ilvD* promoter region (pGY*ilvD*^P^::*lux*) or B) the complete *brnQ1* promoter region (pGY*brnQ1*::*lux*) was pre-grown in complete CDM to mid-exponential phase and then sub-cultured into either complete CDM (284 μM, 684 μM Leu, 684 μM Val) or CDM with limiting/excess concentrations of BCAAs, as indicated. Luminescence (left axis, open shapes) and OD_600nm_ (right axis, filled shapes) were read hourly. Data are the mean +/- SD of three biological replicates.

We selected the CodY-regulated *brnQ1* gene as a representative metabolic gene [7–9], and we modified the luminescent reporter experiment slightly, such that instead of limiting BCAAs, which can alter growth, we added back excess BCAAs and examined whether the addition of excess BCAAs has repressive effects on CodY target gene expression. We first confirmed the effect of excess BCAAs on the *ilvD* promoter. Indeed, we observed that excess amounts of Ile in the growth medium repressed *ilvD* promoter activity (**Fig 10A**), whereas excess Leu had no effect (**Fig 10B**) and, intriguingly, excess Val had the opposite effect of increasing promoter activity (**Fig 10C**). We repeated this experiment with a *lux* promoter:reporter containing the *brnQ1* promoter. Consistent with the *ilvD* promoter:reporter, we observed excess Ile, but not Leu or Val, to have a repressive effect on promoter activity (**Fig 10D–10F**).

**Fig 11:**
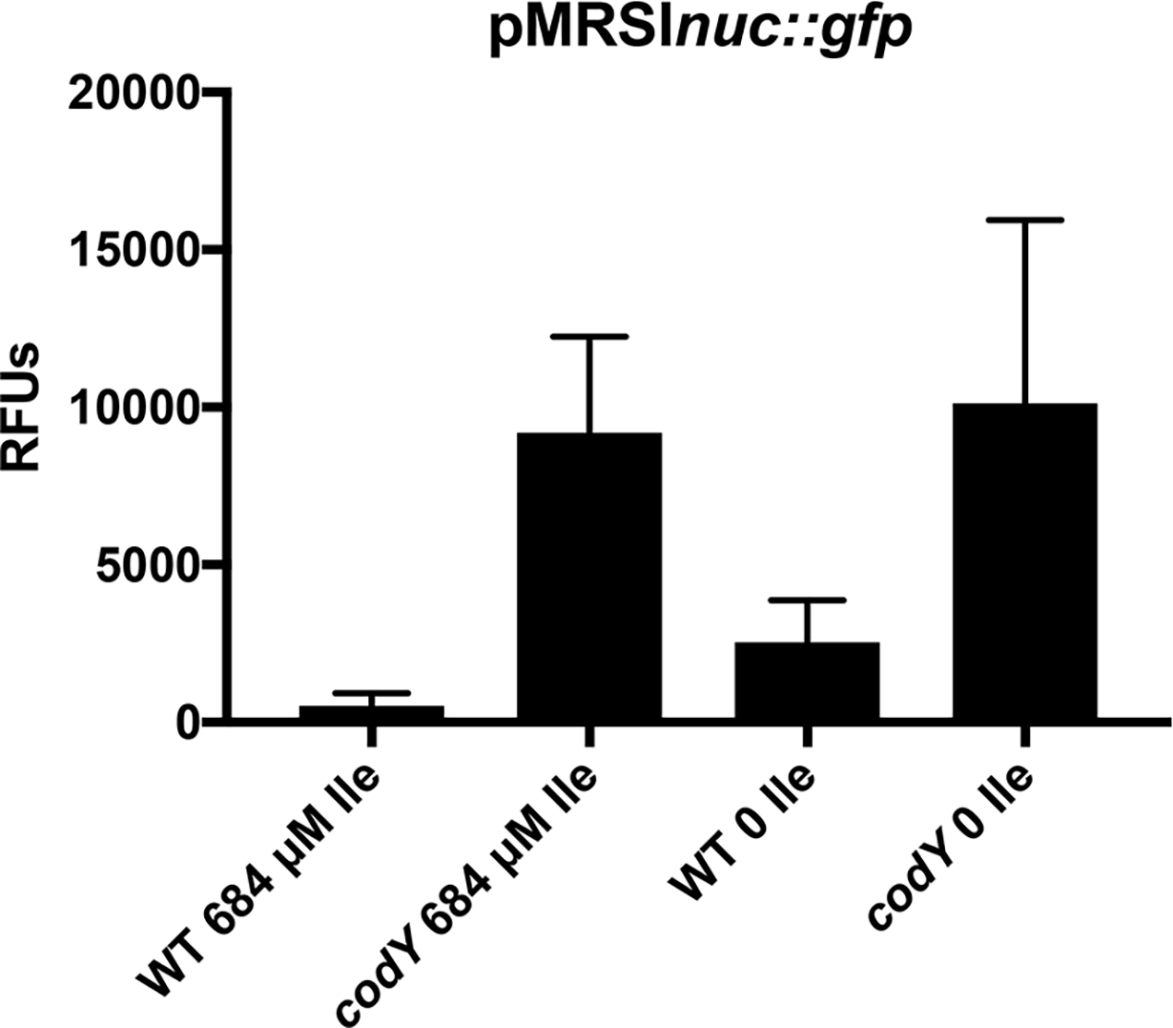
Isoleucine limitation induces *nuc* expression. Strains were pre-grown in complete CDM to exponential phase and then sub-cultured into either complete CDM or CDM lacking Ile, as indicated. Fluorescence values were read when cells achieved mid-exponential phase and were normalized to OD_600nm_. Data are the mean of three biological replicates +/- SEM.

We next investigated whether Ile limitation results in relief of CodY-mediated repression of virulence gene expression, specifically, the secreted factor nuclease [7,9,68]. To do this, we took advantage of a previously constructed *nuc*-*gfp* reporter [7] and measured fluorescence during mid-exponential phase. In agreement with past results, we measured relatively low *nuc-gfp* fluorescence when WT cells were cultured in complete CDM; the fusion was derepressed ~17-fold in *codY* null mutant cells in the same medium (**Fig 11**). When WT cells were grown in CDM lacking Ile, *nuc-gfp* fluorescence increased ~5-fold over that observed in WT cells grown in CDM with excess Ile. Compared with complete CDM, we measured essentially the same amount of *nuc-gfp* fusion fluorescence in *codY* null mutant cells when Ile was omitted from the medium. Thus, Ile limitation results in a partial derepression of *nuc-gfp* in a CodY-dependent manner. Together, these data suggest that the role of Ile in regulating CodY activity is not unique to the *ilvD* promoter.

## Discussion

**Fig 12:**
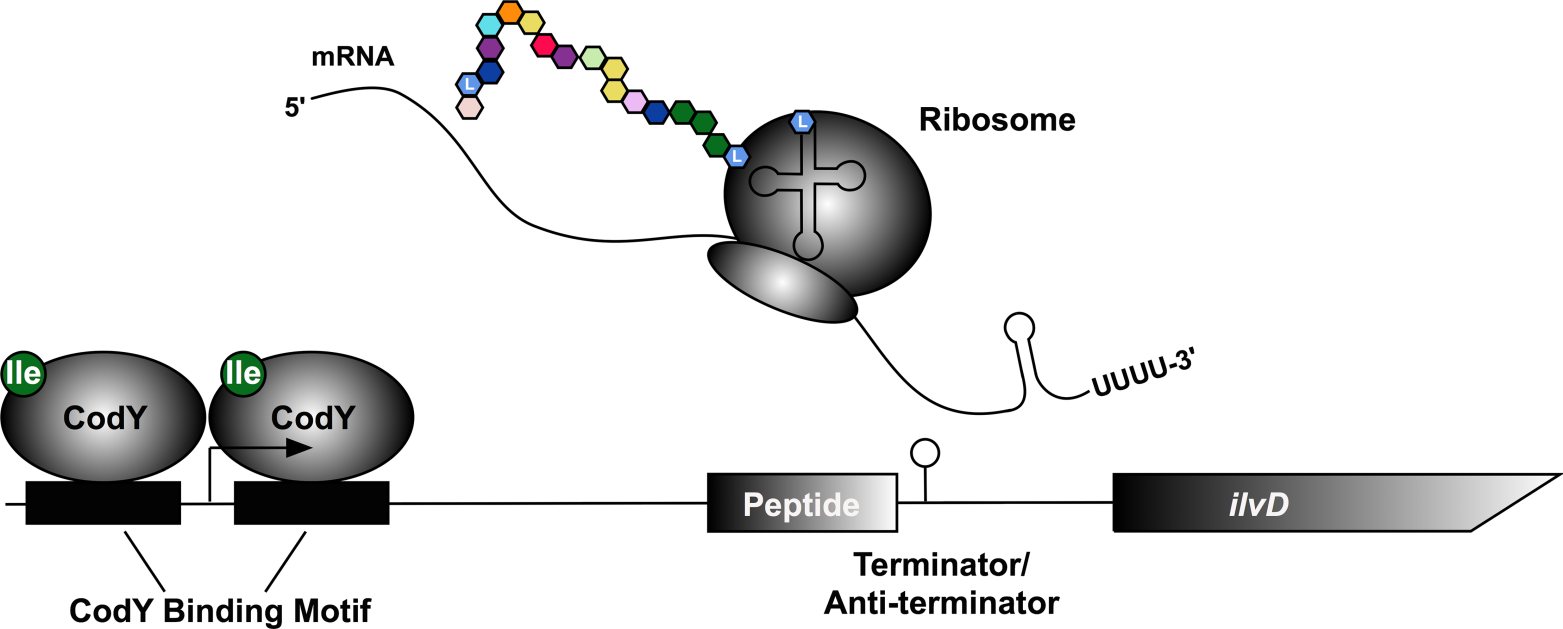
Model of mechanisms regulating *ilv*-*leu* expression. Transcription of the *ilv*-*leu* operon is repressed by CodY. In the presence of Ile, CodY binds and represses transcription. As Ile is depleted, CodY becomes inactive and expression of the operon is induced. As the operon is transcribed, the ribosome beings translating the open reading frame (ORF) upstream of *ilvD*. The ORF is rich in Ile and Leu and will stall if cells are depleted of either tRNA. Stalling of the ribosome prevents formation of the terminator hairpin, allowing transcription of the operon to proceed. When there is sufficient Ile/Leu tRNA, the ORF is translated and the terminator hairpin forms, terminating transcription.

In this study, we sought to determine the mechanisms by which each BCAA regulates expression of the *ilv-leu* operon to explain the unique growth phenotypes of *S*. *aureus* upon depletion of each of Ile, Leu and Val. By selecting for genetic variants of *S*. *aureus* that grew rapidly in the absence of an exogenous source of Val, we characterized two classes of mutations that relieve repression of the *ilv*-*leu* operon; mutations in the transcriptional repressor CodY and mutations in the region upstream of *ilvD*, the first gene in the ILV biosynthetic operon. We demonstrate that CodY activity is predominantly regulated by Ile availability during growth, an unexpected finding given that all three BCAAs activate CodY:DNA binding *in vitro* (**S4 Fig**). Bioinformatic analysis revealed that the region upstream of the *ilvD* coding sequence contains a highly-conserved attenuator peptide that is rich in Leu codons and, therefore, presumably controls transcriptional read-through in response to Leu availability. This is supported by experimental evidence demonstrating that *ilvD* promoter activity increases in response to i) Leu depletion, and ii) mutations in the attenuator peptide. Therefore, Ile and Leu each regulate expression of the *ilv*-*leu* operon through unique mechanisms (summarized in **Fig 12**).

The primary reservoir of *S*. *aureus* is the anterior nares. That Ile was not detected in human nasal secretions [69] lends support to the idea that Ile deprivation is perceived by *S*. *aureus in vivo* and is a signal, via CodY, to upregulate ILV synthesis that would presumably aid in bacterial survival in at least this niche. A predominant role for Ile in regulating CodY activity during growth has also been observed in another *S*. *aureus* strain [8], as well as other species, including *B*. *subtilis* [12], *L*. *lactis* [57], and *L*. *monocytogenes* [70]. Since CodY has been linked with virulence factor expression in *S*. *aureus* [7,8,71–75], including *nuc* as demonstrated in this study, it will be important to determine whether Ile is the predominant BCAA to modulate CodY activity on additional target genes, including virulence genes. It is also noteworthy that we demonstrated an important link between the BrnQ2 transporter, but not BrnQ1 or BcaP, and Ile availability to CodY activity. BrnQ transporters exist in other organisms, yet none function, as BrnQ2 does, as a dedicated Ile-transporter [32,34,35]. This suggests that BrnQ2 could provide an advantage to the adaptation of *S*. *aureus* to Ile-depleted environments. The fact that mutations in CodY are selected for when *S*. *aureus* is grown in the absence of exogenous Val supports the notion that Val contributes minimally to regulating CodY activity, at least on the *ilvD* and *brnQ1* promoters, during growth.

In addition to *trans* regulation, via CodY, of *ilv*-*leu* operon expression in *S*. *aureus*, we identified a *cis*-dependent mode of regulation of operon expression, via an attenuator. Attenuation regulates BCAA biosynthesis in *E*. *coli* and *S*. *enterica* [42–44], and predicted attenuators can be found in various Gram-negative and Gram-positive bacteria [62–64,76]. In support of attenuation regulation of *ilv*-*leu* in *S*. *aureus*, one of the mutations we identified in our screen occurs in the predicted leader peptide and changes a Leu codon to a stop codon, which we predict would reduce Leu-dependent repression. The leader peptide also contains three Ile, suggesting that the level of uncharged tRNA^Ile^ also regulates *ilv*-*leu* expression. In support of this, expression of the *ilv*-*leu* operon is increased upon exposure to mupirocin, an antibiotic that binds to isoleucyl-tRNA synthetase and blocks the charging of tRNA^Ile^ [77]. CodY appears to be the dominant mechanism of repression, since *S*. *aureus* exhibits a growth delay in media lacking Leu, but not Ile, suggesting that Leu deprivation alone is not sufficient to fully relieve repression. Two additional CodY binding regions have been identified in the *ilv-leu* operon [9], and therefore transcription of downstream genes in the operon would occur in a *codY* mutant, bypassing transcriptional termination at the *ilvD* leader. Alternatively, CodY repression could block further transcription upon relief of attenuation, resulting in shorter transcripts. Notably, these conditions did not select for mutations in other previously described regulators of the *ilv-leu* operon, such as Gcp and YeaZ [55,56]. Since Gcp and YeaZ are essential genes in *S*. *aureus*, we did not expect to isolate mutations in these genes, however, we cannot rule out the possibility that the mutations in the attenuator region upstream of the *ilvD* coding region reduce binding of YeaZ [56].

Our model predicts that expression of the *ilv*-*leu* operon would be de-repressed upon Ile and Leu deprivation, yet *S*. *aureus* exhibits a significant growth lag in the absence of all three BCAAs. One possible explanation for this observation is potential allosteric regulation of the BCAA biosynthetic enzymes. The last gene in the *ilv*-*leu* operon, *ilvA*, encodes a threonine deaminase (TD), which catalyzes the first step in Ile synthesis by converting threonine to α-ketobuytrate. The *E*. *coli* TD enzyme is inhibited by Ile and activated by Val [78–80,66]. The *B*. *subtilis* TD enzyme is inhibited by Ile and it is proposed that Val activates TD in the presence of Ile and inhibits TD at high concentrations [67]. If TD activity in *S*. *aureus* is most efficient in the presence of Val, it follows that Ile synthesis would be reduced in the absence of Val. This could explain the absence of growth in media lacking Ile and Val. It would be of interest to investigate whether TD in *S*. *aureus* is similarly subject to allosteric regulation. Given the multiple physiological roles of BCAAs, another possibility is that simultaneous removal of all three BCAAs imparts enhanced stress on *S*. *aureus* compared to when the amino acids are omitted alone. In support of this, we demonstrated that in the absence of Leu and Val acquisition, the *S*. *aureus* membrane lacks Leu- and Val-derived iso-fatty acids, but this loss is compensated for by higher incorporation of Ile-derived iso-fatty acids [25]. Perhaps in the absence of all three BCAAs, such compensatory mechanisms are not achievable. Whatever the mechanism, it is evident that environments where Val is limited or absent poses a challenge to *S*. *aureus* and suggests that Val transport is critical for its growth. Indeed, we have previously shown that BCAA transporters BrnQ1 and BcaP, the only *S*. *aureus* transporters for Val, are required for *S*. *aureus* growth *in vivo* [24,25].

Altogether, this study details the molecular mechanisms regulating BCAA biosynthesis in *S*. *aureus* and uncovers environments where *S*. *aureus* engages in BCAA biosynthesis. In doing so, we reveal a predominant role for Ile in regulating CodY activity on the *ilvD* and *brnQ1* promoter. Given the role of CodY in additionally regulating virulence genes, our data support the hypothesis that environmental availability of Ile is an important regulatory cue for *S*. *aureus* adaptation to nutrient limitation and virulence gene expression.

## Materials and methods

### Growth conditions

All strains and plasmids used in this study are described in **Table 3**. Methicillin-resistant *S*. *aureus* (MRSA) pulsed-field gel electrophoresis type USA300 LAC that has been cured of pUSA03, a plasmid conferring macrolide and lincosamide resistance, was used in all experiments as the wild-type (WT) strain. *S*. *aureus* strains were grown in either tryptic soy broth (TSB) (EMD Millipore, Billerica, MA) or in a chemically defined medium (CDM), described previously [24]. Final concentrations of Ile, Leu and Val in complete CDM were 228 μM, 684 μM, and 684 μM, respectively. Final concentrations were adjusted to 10% of their concentration in complete CDM in some experiments, as indicated. For growth experiments in TSB, *S*. *aureus* strains were pre-cultured in TSB until mid-exponential phase was reached, and then sub-cultured into fresh TSB to a starting optical density (OD_600_) of 0.01. For growth experiments in CDM, *S*. *aureus* strains were pre-cultured in CDM until mid-exponential phase was reached, and then sub-cultured into fresh CDM to a starting OD_600_ of 0.05 in either complete CDM or CDM where BCAA concentrations were limited or omitted, as indicated. Growth curves were performed in 100-well plates containing 200 μL/well of media and were read using the Bioscreen C visible spectrophotometer (Growth Curves USA; Piscataway, NJ). End point growth assays were performed in tubes with a 7:1 v/v tube:media ratio. All growth experiments were performed at 37°C with shaking. Growth media were supplemented with chloramphenicol (10 μg mL^-1^), ampicillin (100 μg mL^-1^), or erythromycin (3 μg mL^-1^), where required.

**Table 3:** Strains and plasmids.

Strain or Plasmid	Description^a^	Source or reference
**Strains**		
***S*. *aureus***		
USA300	USA300 LAC cured of antibiotic resistance plasmid	Heinrichs lab stock
RN4220	r_K_^-^ m_K_^+^; capable of accepting foreign DNA	[81]
H3001	USA300 *codY*::ϕNΣ; Em^R^	[82]
H2568	USA300 Δ*brnQ1*	[24]
H2563	USA300 Δ*brnQ2*	[24]
H3386	USA300 Δ*bcaP*	This study
H3584	USA300 Δ*brnQ1*Δ*bcaP*	This study
SRB687	USA300 LAC cured of antibiotic resistance plasmid	A. Horswill
SRB746	USA300 Δ*codY*::*ermC*	[7]
SRB837	USA300 /pRMS1-*nuc bla cat nuc-gfp*	[83]
SRB838	USA300 Δ*codY*::*ermC* /pRMS1-*nuc bla cat nuc-gfp*	[83]
***E*. *coli***		
DH5α	F^-^ ϕ80d*lacZ*ΔM15 *recA1 endA1 gyrA96 thi-1 hsdR17*(r_K_^-^ m_K_^-^) *supE44 relA1 deoR* Δ(*lacZyA-argF)U169 phoA*	Promega
**Plasmids**		
pRMC2	Anhydrotetracycline-inducible expression vector; Ap^R^ in *E*. *coli*; Cm^r^ in *S*. *aureus*	[84]
p*codY*	pRMC2 containing *codY*; Cm^R^	This study
pGY*lux*	Vector harboring promoterless *luxABCDE* operon; Cm^R^	[85]
pGY*ilvD*^WT^::*lux*	*Lux* reporter vector with *ilvD* promoter from WT USA300; Cm^R^	This study
pGY*ilvD*^ValS1^::*lux*	*Lux* reporter vector with *ilvD* promoter mutated to contain the Val^Sup^-1 SNP; Cm^R^	This study
pGY*ilvD*^ValS7^::*lux*	*Lux* reporter vector with *ilvD* promoter mutated to contain the Val^Sup^-7 SNP; Cm^R^	This study
pGY*ilvD*^ValS9^::*lux*	*Lux* reporter vector with *ilvD* promoter mutated to contain the Val^Sup^-9 SNP; Cm^R^	This study
pGY*ilvD*^P^::*lux*	*Lux* reporter vector with only the CodY binding motifs in the *ilvD* promoter; Cm^R^	This study
pGY*ilvD*^C^::*lux*	*Lux* reporter vector with the *ilvD* promoter from WT USA300; Cm^R^	This study
pRMS1-*nuc*	GFP reporter vector with *nuc* promoter from WT UAMS-1; Cm^R^	[7]

^a^Abbreviations: Em^R^, Ap^R^, Cm^R^, designate resistance to erythromycin, ampicillin and chloramphenicol respectively.

### Mutagenesis and construction of plasmids

Deletion of *bcaP* was constructed using the pKOR1 plasmid as described previously [24]. Primer sequences were based on the published USA300 FPR3757 genome and are displayed in **Table 4**. The *bcaP* deletion was introduced into the markerless *brnQ1* deletion mutant, described previously [24]. The pGY*lux* vectors were constructed using primers described in **Table 4**. The pGY*lux* plasmid is derived from a low copy plasmid (5 copies/cell) [85], and we estimated 20 copies/cell in our experiments. *lux* plasmids were further used as templates for site-directed mutagenesis, using primers described in **Table 4**. Briefly, PCR reactions containing the Phusion High-Fidelity DNA Polymerase (ThermoFisher, Waltham, MA) were set up such that half of the reaction mixture contained the forward primer and the remaining half contained the reverse primer. These reactions proceeded for 3 cycles of 98°C for 10 s, 60°C for 30 s, and 72°C for 12 min. After 3 cycles, the forward and reverse primer reactions were mixed together and the reactions proceeded for an additional 17 cycles. Plasmids were treated with *Dpn*I (New England Biolabs, Ipswich MA) for 1 hr at 37°C and were then transformed into *E*. *coli* DH5α. Mutations were confirmed by PCR. All plasmids were first constructed in *E*. *coli* DH5α and subsequently electroporated into the restriction-defective *S*. *aureus* strain, RN4220, prior to electroporation into the desired strain.

**Table 4:** Oligonucleotides used in this study.

Oligonucleotides ^a^	Sequence (5’-3’)
*bcaP* Ups F *bcaP* Ups R	GGGGACAAGTTTGTACAAAAAAGCAGGCTCAGTCTTCGTATTCACCTGC CTTCCCATAAACTTTCCTCC For generating upstream arm for *bcaP* deletion
*bcaP* Dwn F *bcaP* Dwn R	5’ /Phos/ ACGTAGCTGAATACCACCC GGGGACCACTTTGTACAAGAAAGCTGGGTTGTACCTGCTGACGAAGTAG For generating downstream arm for *bcaP* deletion
*ilvD*^*C*^ F *ilvD*^*C*^ R	GATCCCCGGGACCTGCTCCTAAATCTCCG GATCGTCGACACTTCTTGCTGGTGCTTGG For cloning *ilvD* 5’UTR into pGY*lux*
*ilvD*^P^ F *ilvD*^P^R	GATCCCCGGGGTACGTCTTACACCAAG GATCGTCGACAGTTGTCGGTTGATGTTC For cloning partial *ilvD* 5’UTR into pGY*lux*
*ilvD*^ValS-1^ F *ilvD*^ValS-1^ R	CAA ATA TTA TTA TTT TAT aAT ACT CTT TAG GAC TCG CGA GTC CTA AAG AGT ATt ATA AAA TAA TAA TAT TTG For site directed mutagenesis of pGY*lux*::*ilvD*
*ilvD*^ValS-7^ F *ilvD*^ValS-7^ R	CTA AAC GCT TTA AGT CaT ATT TCT GTT TGA ATG CAT TCA AAC AGA AAT AtG ACT TAA AGC GTT TAG For site directed mutagenesis of pGY*lux*::*ilvD*
*ilvD*^ValS-9^ F *ilvD*^ValS-9^ R	CTA AAC GCT TTA AGc CCT ATT TCT GTT TG CAA ACA GAA ATA GGg CTT AAA GCG TTT AG For site directed mutagenesis of pGY*lux*::*ilvD*
*codY* F *codY* R	GATCGGTACCCCGAATGCAGTTGTAGATATTACC GATCGAGGCTCTTATGTCCCAGACTCATCGAC For cloning *codY* into pRMC2
*codY* Seq F *codY* Seq R	GCAATTACTCGCTTAGCTGAG GTGTGTATTGGCTTTATAGCCG For target directed sequencing of *codY*
*ilvD* qPCR F *ilvD* qPCR R	GCTATCTTTTGCTCTGGTGG AGGGCAGGCATTTTGTTCC For qPCR of *ilvD*
*ilvC* qPCR F *ilvC* qPCR R	CAAGATGTAAAAACGGACGC GTCAAAAGAACGACCTGGG For qPCR of *ilvC*
oAK031	6-FAM/ATCCATTGTTCAATCGTATC
oNW025	GAAGTTGTCGGTTGATGTTC generate *ilvD266p* for EMSA

^a^ All primer sequences, except oAK031 and oNW025 (based on UAMS-1), are based on the USA300 FPR3757 genome; restriction sites are underlined; nucleotide mutated in site directed mutagenesis is indicate in lower case.

### Selection of Val^Sup^ mutants and whole genome sequencing

To select for genetic mutations that permit adaptation to growth in media lacking Val, twelve independent colonies of WT *S*. *aureus* were grown in complete CDM to mid-exponential phase and sub-cultured into CDM lacking Val. Recovered cells were harvested and plated onto TSB agar and grown overnight at 37°C. Isolated colonies were grown in complete CDM to mid-exponential phase and sub-cultured into CDM lacking Val to confirm the occurrence of a heritable mutation. Genomic DNA was isolated from all twelve mutants, referred to as Val^Sup^-mutants, as well as from two biological replicates of our laboratory WT USA300, using the Invitrogen PureLink Genomic DNA Preparation Kit (ThermoFisher Scientific, Boston MA) per the manufacturer’s instructions. Primers used for the targeting sequencing of the *ilvD* promoter and the *codY* gene are listed in **Table 4**. Samples were sent to the London Regional Genomics Center for sequencing on the MiSeq platform. Libraries were prepared using the Nextera XT DNA Library Preparation kit (Illumina, San Diego, CA). 150 bp reads were mapped to the USA300 FPR3757 (NC_007793.1) genome using the BWA-MEM aligner [86] and variants were determined using SAMtools [87].

### TCA precipitation of proteins and SDS-PAGE

Strains were pre-grown in TSB to mid-exponential phase and then sub-cultured into TSB to a starting OD_600_ of 0.01 and grown overnight. The OD_600_ of stationary phase cultures were determined and a supernatant volume equivalent to 5 OD units was harvested and incubated with trichloroacetic acid (TCA) (Sigma-Aldrich, St. Louis, MO) at a final concentration of 20% overnight at 4°C. Precipitated protein samples were dissolved, run on a 12% acrylamide gel and stained with Coomassie-Blue.

### *lux* reporter assays

Kinetic *lux* reporter experiments were performed in flat, clear-bottom 96-well white plates (Thermo Fisher Scientific) and read using a BioTek Synergy H4 Hybrid Multi-Mode Microplate Reader (BioTek Instruments Inc, Winooski, VT). Pre-cultures were inoculated into either complete or limited CDM to a starting OD_600_ of 0.01 in 200 μL/well. Luminescence and OD_600_ values were read at hourly intervals. For end-point *lux* reporter experiments, pre-cultures were sub-cultured into either complete or limited CDM to a starting OD_600_ of 0.05 in tubes with a 7:1 tube:media ratio. At hourly intervals, aliquots of 200 μL were transferred to flat, clear-bottom 96-well white plates (Thermo Fisher Scientific) and luminescence and OD_600_ values were read. Samples of strains containing the *lux* construct with the complete *ilvD* promoter were supplemented with 0.1% (v/v) decanal in 40% ethanol and luminescence was measured immediately. Data presented are the relative light unit (RLU) values normalized to the OD_600_ of the sample when the cultures reached mid-exponential phase (OD_600_ 0.6–0.8). Data were analyzed by one-way ANOVA with Dunnet’s multiple comparison test relative to the control sample in GraphPad Prism Version 7.0b.

### RT-qPCR

RNA was isolated from cells grown to mid-exponential phase (OD_600_ of 0.6–0.8) in complete CDM using the Aurum Total RNA Mini Kit (Bio-Rad; Hercules, CA) per the manufacturer’s instructions. RNA (500 ng) was reverse transcribed using SuperScript II (Invitrogen, Carlsbad, CA) per the manufacturer’s instructions using 500 μg mL^-1^ of random hexamers. cDNA was PCR-amplified using SensiFAST SYBR No-ROX Kit (Bioline, Taunton, MA). Data were normalized to expression of the *rpoB* reference gene, and analyzed by an unpaired student’s *t*-test in GraphPad Prism Version 7.0b. Primers used are listed in **Table 4**.

### Cloning, expression, and purification of recombinant codY protein

The *codY* ORF (QV15_05910) was amplified from *S*. *aureus* strain UAMS-1 using oligonucleotides oKM1 and oSRB410. The PCR fragment was purified and subjected to a second round of PCR using oKM1 and oSRB411 to append a Tobacco Etch Virus (TEV) protease cleavage sequence followed by six histidine (CAT) codons and a TAA stop codon. The 830-nt fragment was digested with SacI/SphI and ligated to the same sites of pBAD30 [88]. The resulting plasmid was introduced into *E*. *coli* DH5α. CodY-His_6_ was overproduced by growing the strain carrying the plasmid in LB at 37°C until mid-exponential phase (OD_600_ ~0.3). At this time, L-(+)-arabinose was added to a final concentration of 0.2% (w/v). After four hours of induction at 37°C, cells were pelleted by centrifugation (8,610 *x g* at 4°C) and frozen at -80°C. The cells were thawed, resuspended in Buffer A (20 mM Tris-Cl [pH 7.9], 500 mM NaCl, 5 mM imidazole, 5% [v/v] glycerol) supplemented with 0.1% (v/v) nonidet P-40 and 1 mM phenylmethylsulfonyl fluoride (PMSF), and lysed by sonication. CodY-His_6_ protein was purified from clarified soluble extracts using a computer-controlled ÄKTAPrime plus FPLC system equipped with a His-Trap FF column (GE Healthcare Life Sciences) using a linear gradient elution with Buffer B (20 mM Tris-Cl [pH 7.9], 500 mM NaCl, 685 mM imidazole, 5% [v/v] glycerol). Fractions containing CodY-His_6_ protein were pooled and supplemented with glycerol to 50% (v/v) and stored at -20°C.

### Electrophoretic mobility shift assays (EMSAs)

A 266-bp fragment (*ilvD*_*266*_*p*^*+*^) spanning -131 to +134 relative to the annotated *ilvD* transcriptional start site in *S*. *aureus* UAMS-1 [9] was synthesized by PCR using primers oNW025 and oAK031 (**Table 4**), simultaneously incorporating a 6-carboxyfluorescein (FAM)-label. EMSAs were performed with purified CodY-His_6_ protein and FAM-labeled *ilvD*_*266*_*p*^*+*^ fragment in binding buffer (20 mM Tris-Cl [pH 8.0], 50 mM KCl, 2 mM MgCl_2_, 5% [v/v] glycerol, 0.05% [v/v] Nonidet P-40, 1 mM dithiothritol [DTT], 0.025 mg ml^-1^ salmon sperm DNA). Samples (20 μl) containing various amounts of CodY-His_6_, 200 fmol of 6-FAM-labeled DNA fragment, 2 mM GTP, and 10 mM of the indicated BCAA(s) were incubated for 20 min at 25°C in a thermomixer (Eppendorf) with moderate agitation (250 rpm). The samples were separated on 8% non-denaturing 35 mM HEPES (pH 7.4)-43 mM imidazole-10 mM BCAA polyacrylamide gels for 40 minutes at 200 V. Fluorescent DNA fragments were detected and quantified using a computer-controlled ImageQuant LAS 4000 biomolecular imager (GE Healthcare Life Sciences) using a SYBR filter set. Quantitative analysis of CodY binding to *ilvD*_*266*_*p*^*+*^ was performed using ImageJ software [89]. Since the binding curves appeared to have a sigmoidal shape, the data from two independent experiments were fitted to the Hill equation Θ = C^h^/(C^h^ + *K*_0.5_^h^) using Prism (ver. 7; GraphPad Software). In this equation, Θ is the fraction of bound DNA, C is the concentration of CodY, *K*_0.5_ is the binding constant, and h is the Hill coefficient. *K*_0.5_ and h shown are from fitted data where *r*^*2*^ > 0.96.

### *nuc-gfp* reporter assays

Strains were grown overnight in CDM complete, then sub-cultured the next morning in CDM complete to a starting OD_600_ of 0.05 in 125 ml DeLong shake flasks (5:1 flask:medium ratio). Incubation was performed in an Innovo orbital shaking water bath (New Brunswick) with vigorous agitation (280 rpm). At an OD_600_ of 0.8, cells were pelleted and resuspended in either CDM complete or CDM lacking isoleucine to an initial OD_600_ of ~0.05. When cells reached mid-exponential phase (OD_600_ of 0.4–0.5), a 1-mL sample was removed, washed once with phosphate buffered saline (PBS), and resuspended in PBS to minimize background fluorescence from the medium. Fluorescence was measured using a computer-controlled Tecan Infinite F200 Pro plate reader equipped with 485 nm excitation and 535 nm emission filters. GFP signal acquisition parameters were kept constant throughout the experiment (gain, 49%; flash number, 10; integration time, 40 μs; lag time, 0 μs; settle time, 0 ms). Data are presented as relative fluorescence units (RFUs) after subtracting the fluorescence from USA300 LAC (lacking the GFP reporter plasmid) and dividing by OD_600_ to correct for cell density.

### Bioinformatics

Putative terminator structures in the *ilvD* 5’UTR were identified using the predictive software RibEx [90] and RNAfold [91]. Mfold [92] was used to identify putative antiterminator sequences. RNAfold and Mfold were used to search for conserved T-box riboswitch features. T-box riboswitch multiple sequence alignments were generated with predicted T-box riboswitch sequences in *S*. *aureus* subsp. *aureus* N315 (NC_002745.2) that were annotated in the Rfam database [93] and the *ilvB* T-box riboswitch from *B*. *subtilis* (NC_000964.3) using MUSCLE [94] with default parameters. The alignments were manually adjusted in JalView [95] with insight gained from experimentally characterized *S*. *aureus* T-box leaders: *glyS*, *ileS* and *metI*. The putative *S*. *aureus ilvD* leader was then added to the finished alignment using MAFFT [96]. The peptide multiple sequence alignments were generated by extracting the top 15 BLAST results for *ilvD* leaders from different staphylococci, translating the ORFs, and aligning the peptide sequences using MUSCLE [94]. The *ilvD* 5’UTR from USA300 FPR3757 was aligned to 168 *S*. *aureus* complete genomes using BLAST.

## Supporting information

S1 Fig Secreted protein profiles of Val^Sup^-mutants with WT and *codY* mutant. Strains were pre-grown in TSB to mid-exponential phase, then sub-cultured into TSB for 16 hr. Supernatants were collected and proteins were precipitated using TCA. Protein samples were normalized to the equivalent of 5 ODs and run on a 12% SDS-PAGE gel.
(TIF)

S2 Fig Sequence alignment of T-box riboswitches. Sequences of all annotated *S*. *aureus* T-boxes (based on the *S*. *aureus* subsp. *aureus* N315 genome NC_002745.2) and the *B*. *subtilis ilvB* T-box (NC_000964.3), labelled by regulated gene, were analyzed. Key features analyzed and annotated above the alignment are the AG Bulge (AGVGA-box), Distal Loop (GNUG-box); and the Specifier Loop (GAA…XXXA) where XXX represents the tRNA codon.
(TIF)

S3 Fig Nucleotide sequence alignment and RNA secondary structure prediction of the attenuator and terminator region in the region upstream of *ilvD* across staphylococcal species. (A) Multiple sequence alignments of the top 15 hits from a BLAST search using the *S*. *aureus ilvD* promoter region were extracted and aligned as described in the methods. Dark blue shading represents conservation above 80%. Coding regions and key structures are labeled above and below the alignment. (B) Secondary structure predictions for the region aligned in the top panel. The start and stop codons are highlighted in green and red, respectively and the alternative pairing regions in the terminator/antiterminator are highlighted in yellow and blue.
(TIF)

S4 Fig Individual BCAAs or a combination of all three BCAAs activate CodY DNA-binding activity *in vitro*. 6-FAM-labeled *ilvD*_*266*_*p*^*+*^ DNA fragment was incubated with increasing amounts of *S*. *aureus* CodY protein in the presence of GTP and A) isoleucine, B) leucine, C) valine, or all three amino acids (ILV). Concentrations of CodY used (nM of monomer) are indicated below each lane. Unbound DNA fragments are indicated by the right-pointing open arrowheads; CodY:*ilvD*_*266*_*p*^*+*^ complexes are indicated by the right-pointing closed arrowheads. Data are representative of at least two independent experiments.
(TIF)

S5 Fig Summary of mutations identified following nucleotide sequence alignment of the region upstream of *ilvD* across *S*. *aureus* strains. The *ilvD* promoter region was aligned across 168 complete *S*. *aureus* genomes. In (A), the location of the mutations is indicated relative to the transcription start site identified by Majercyzk *et al*., 2010 [9]. The number of strains containing the mutation is indicated in brackets. Mutations in green are the SNPs identified in this study. Shown in (B) are the CodY binding sites identified by Majercyzk *et al*., 2010 [9], with the canonical CodY motif in bold and italicized, along with the predicted anti-terminator and terminator sequences. In (C) is listed the currently available strains for which genomes have the identified SNPs that are shown in panel A.
(TIF)
