# Supplementary figures and images for: Repression of branched-chain amino acid synthesis in Staphylococcus aureus is mediated by isoleucine via CodY, and by a leucine-rich attenuator peptide
Source: PLoS Genet. 2018 Jan 22;14(1):e1007159. doi: 10.1371/journal.pgen.1007159 (PMC5794164; doi:10.1371/journal.pgen.1007159)

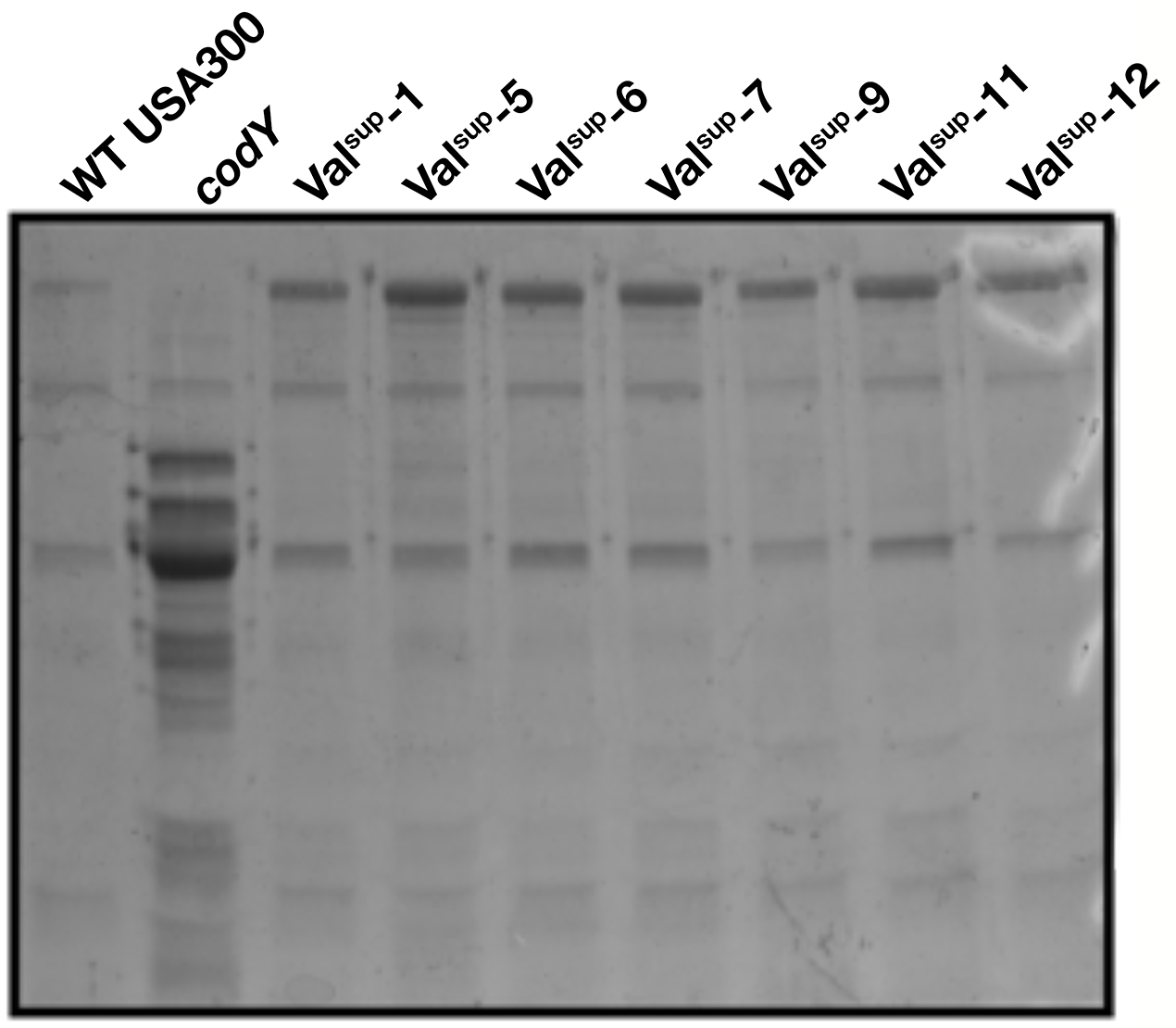

Supplement: S1 Fig — Strains were pre-grown in TSB to mid-exponential phase, then sub-cultured into TSB for 16 hr. Supernatants were collected and proteins were precipitated using TCA. Protein samples were normalized to the equivalent of 5 ODs and run on a 12% SDS-PAGE gel. (TIF) [file pgen.1007159.s001.tif]

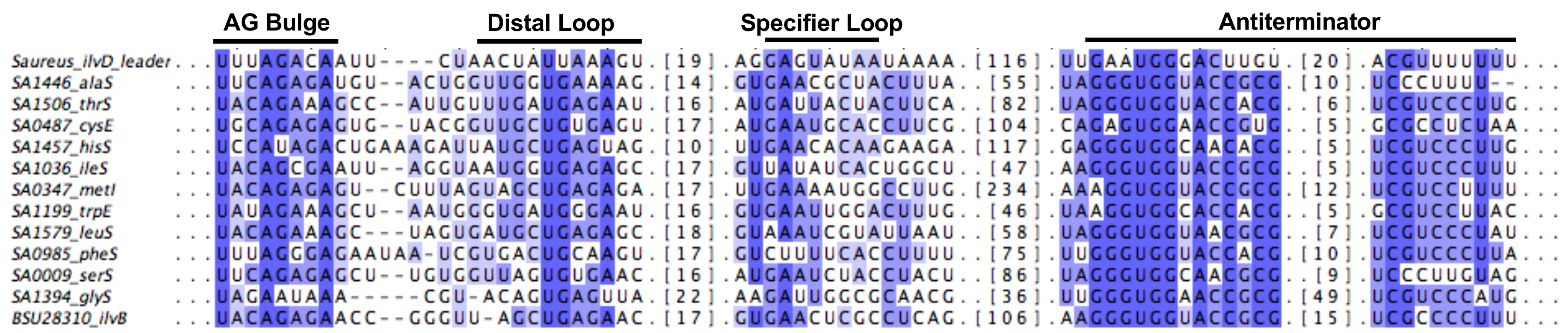

Supplement: S2 Fig — Sequences of all annotated S. aureus T-boxes (based on the S. aureus subsp. aureus N315 genome NC_002745.2) and the B. subtilis ilvB T-box (NC_000964.3), labelled by regulated gene, were analyzed. Key features analyzed and annotated above the alignment are the AG Bulge (AGVGA-box), Distal Loop (GNUG-box); and the Specifier Loop (GAA…XXXA) where XXX represents the tRNA codon. (TIF) [file pgen.1007159.s002.tif]

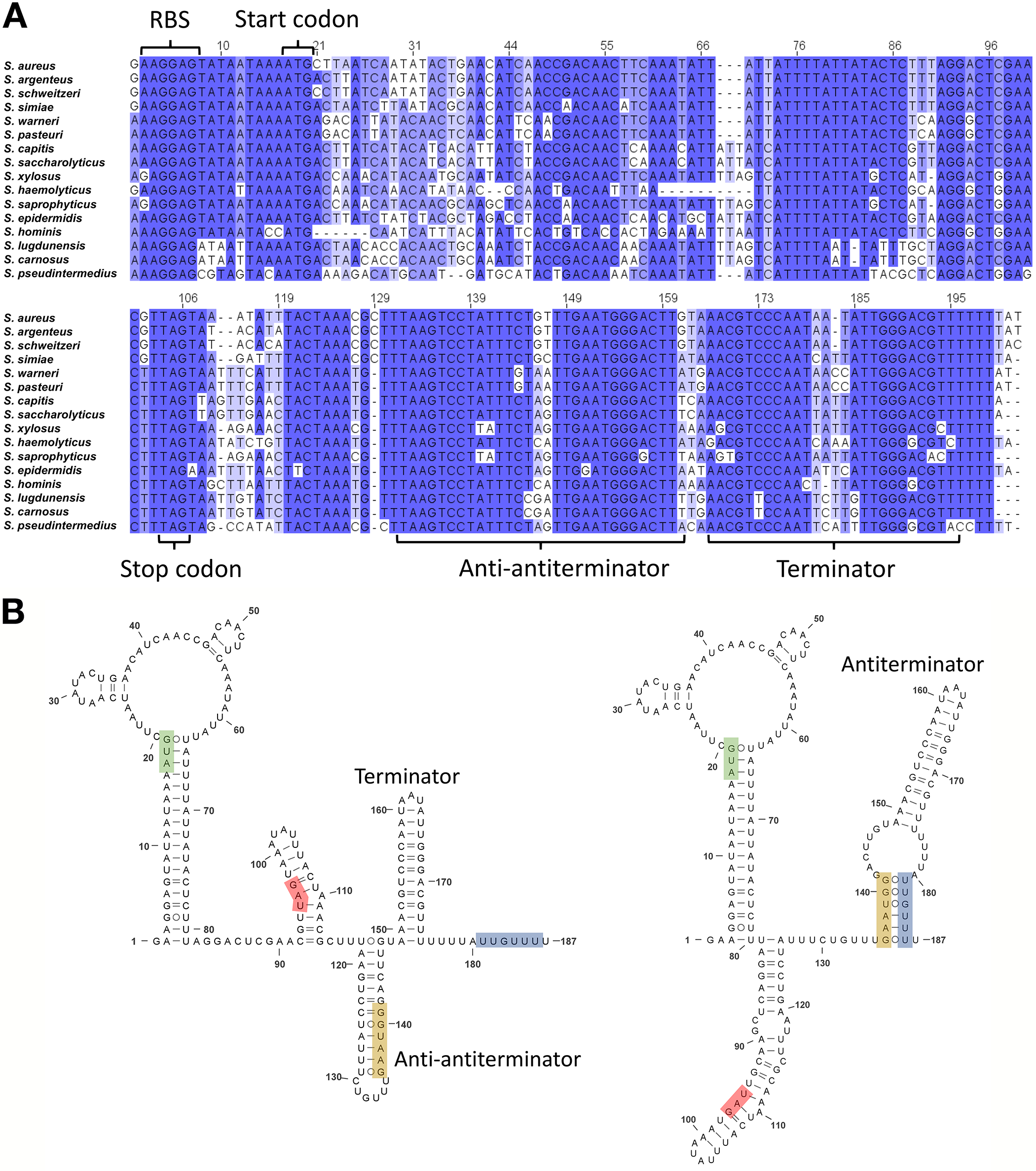

Supplement: S3 Fig — (A) Multiple sequence alignments of the top 15 hits from a BLAST search using the S. aureus ilvD promoter region were extracted and aligned as described in the methods. Dark blue shading represents conservation above 80%. Coding regions and key structures are labeled above and below the alignment. (B) Secondary structure predictions for the region aligned in the top panel. The start and stop codons are highlighted in green and red, respectively and the alternative pairing regions in the terminator/antiterminator are highlighted in yellow and blue. (TIF) [file pgen.1007159.s003.tif]

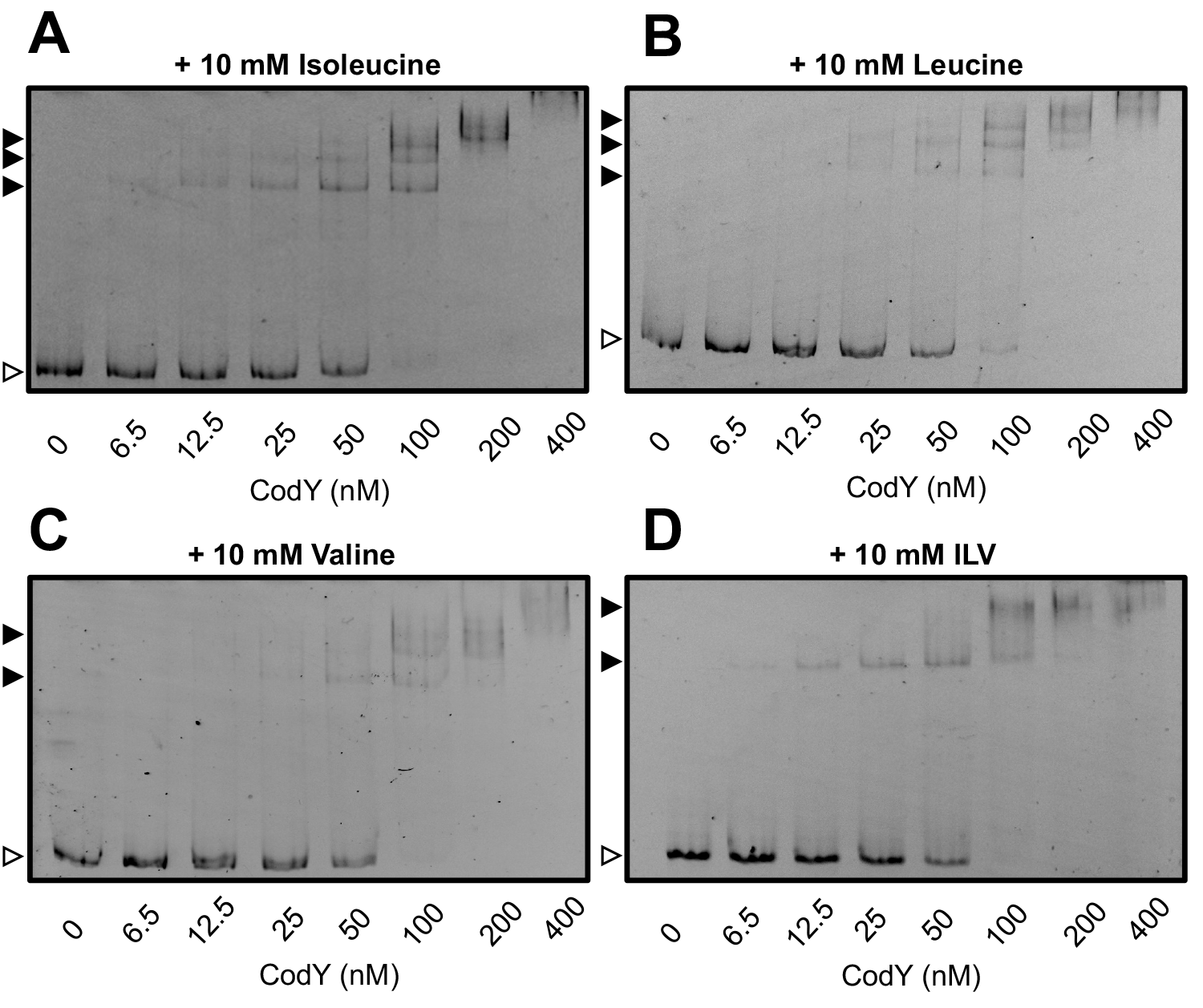

Supplement: S4 Fig — 6-FAM-labeled ilvD266p+ DNA fragment was incubated with increasing amounts of S. aureus CodY protein in the presence of GTP and A) isoleucine, B) leucine, C) valine, or all three amino acids (ILV). Concentrations of CodY used (nM of monomer) are indicated below each lane. Unbound DNA fragments are indicated by the right-pointing open arrowheads; CodY:ilvD266p+ complexes are indicated by the right-pointing closed arrowheads. Data are representative of at least two independent experiments. (TIF) [file pgen.1007159.s004.tif]

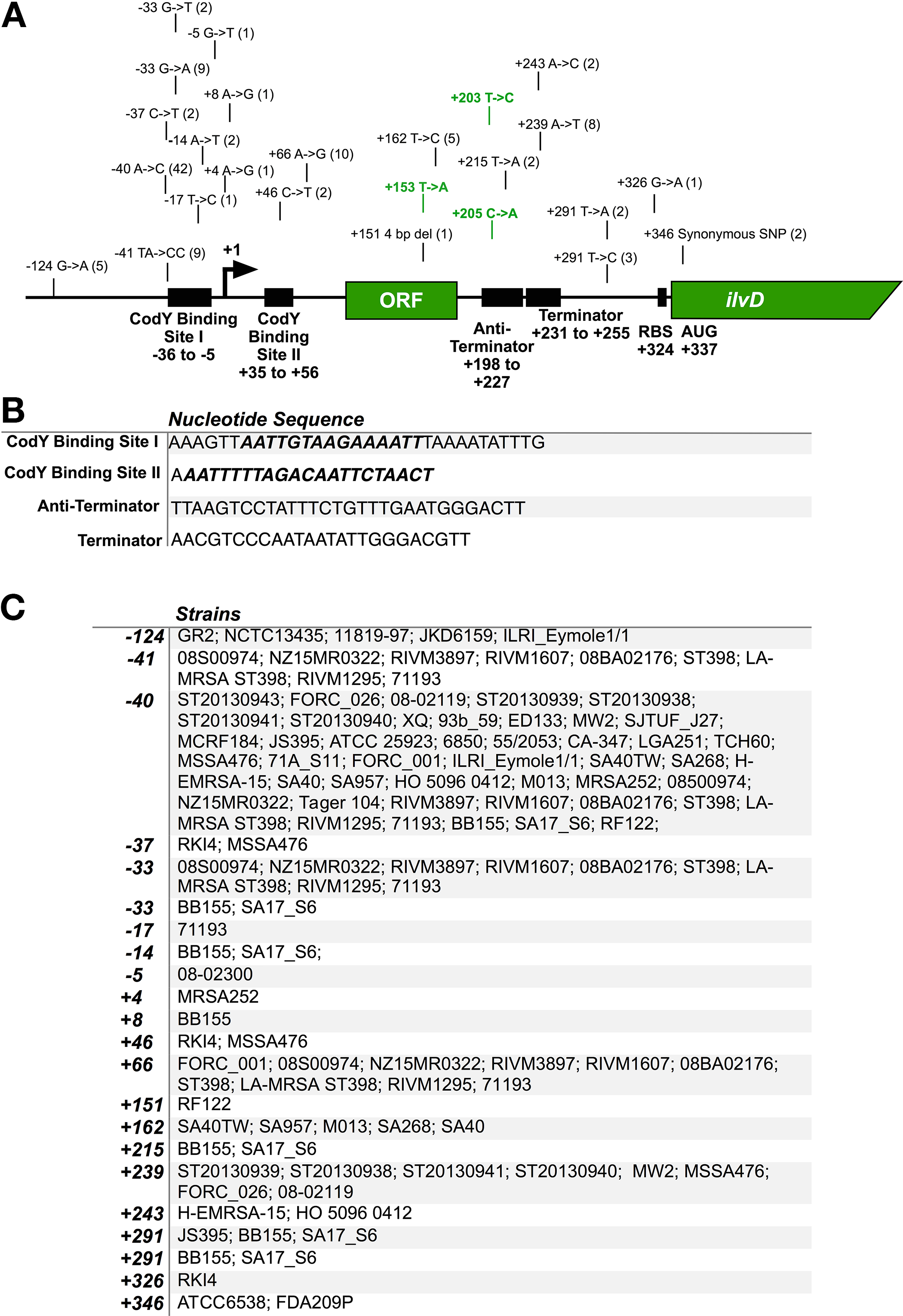

Supplement: S5 Fig — The ilvD promoter region was aligned across 168 complete S. aureus genomes. In (A), the location of the mutations is indicated relative to the transcription start site identified by Majercyzk et al., 2010 [9]. The number of strains containing the mutation is indicated in brackets. Mutations in green are the SNPs identified in this study. Shown in (B) are the CodY binding sites identified by Majercyzk et al., 2010 [9], with the canonical CodY motif in bold and italicized, along with the predicted anti-terminator and terminator sequences. In (C) is listed the currently available strains for which genomes have the identified SNPs that are shown in panel A. (TIF) [file pgen.1007159.s005.tif]
